# Proof of concept of a new plasma complement Factor H from waste plasma fraction

**DOI:** 10.3389/fimmu.2024.1334151

**Published:** 2024-06-06

**Authors:** Filippo Mori, Giancarlo Pascali, Silvia Berra, Alessandra Lazzarotti, Daniele Panetta, Silvia Rocchiccioli, Elisa Ceccherini, Francesco Norelli, Antonio Morlando, Roberta Donadelli, Alberto Clivio, Claudio Farina, Marina Noris, Piero A. Salvadori, Giuseppe Remuzzi

**Affiliations:** ^1^ Research and Innovation, Kedrion Biopharma, Lucca, Italy; ^2^ Biosciences, Australian Nuclear Science and Technology Organisation, Lucas Heights, NSW, Australia; ^3^ School of Chemistry, University of New South Wales, Kensington, NSW, Australia; ^4^ Department of Biomedical and Clinical Sciences (DIBIC), University of Milan, Milan, Italy; ^5^ Istituto di Fisiologia Clinica, Consiglio Nazionale delle Ricerche, Pisa, Italy; ^6^ Istituto di Ricerche Farmacologiche Mario Negri IRCCS, Bergamo, Italy

**Keywords:** concentrated complement factor H (FH), plasma purification, high-resolution dynamic PET, C3 glomerulopathy, membrano proliferative glomerulonephritis (MPGN)

## Abstract

**Introduction:**

Complement factor H (FH) is a major regulator of the complement alternative pathway, its mutations predispose to an uncontrolled activation in the kidney and on blood cells and to secondary C3 deficiency. Plasma exchange has been used to correct for FH deficiency and although the therapeutic potential of purified FH has been suggested by *in vivo* experiments in animal models, a clinical approved FH concentrate is not yet available. We aimed to develop a purification process of FH from a waste fraction rather than whole plasma allowing a more efficient and ethical use of blood and plasma donations.

**Methods:**

Waste fractions from industrial plasma fractionation (pooled human plasma) were analyzed for FH content by ELISA. FH was purified from unused fraction III and its decay acceleration, cofactor, and C3 binding capacity were characterized *in vitro.* Biodistribution was assessed by high-resolution dynamic PET imaging. Finally, the efficacy of the purified FH preparation was tested in the mouse model of C3 glomerulopathy (Cfh−/− mice).

**Results:**

Our purification method resulted in a high yield of highly purified (92,07%), pathogen-safe FH. FH concentrate is intact and fully functional as demonstrated by *in vitro* functional assays. The biodistribution revealed lower renal and liver clearance of human FH in Cfh-/- mice than in wt mice. Treatment of Cfh-/- mice documented its efficacy in limiting C3 activation and promoting the clearance of C3 glomerular deposits.

**Conclusion:**

We developed an efficient and economical system for purifying intact and functional FH, starting from waste material of industrial plasma fractionation. The FH concentrate could therefore constitute possible treatments options of patients with C3 glomerulopathy, particularly for those with FH deficiency, but also for patients with other diseases associated with alternative pathway activation.

## Introduction

Complement factor H (FH) is a 155-kDa soluble glycoprotein produced in the liver and secreted into the circulation at concentrations of 250–600 µg / ml (1). It is a single polypeptide chain with a beads-in-a-string-like structure composed of 20 homologous domains named short consensus repeat (SCR) or complement control protein (CCP) domains (2). It regulates the alternative pathway (AP) of the complement system in the fluid phase as well as on cell surfaces preventing uncontrolled C3 activation and host tissue damage. Specifically, FH accelerates the dissociation of the C3 convertase (C3bBb) and C5 convertase (C3bBbC3b) (decay-accelerating activity), acts as a cofactor to factor I in the inactivation of C3b into iC3b (cofactor activity) and competes with FB in binding to C3b to prevent convertase formation. Through its C-terminal domains SCR19–20, FH binds cell surface glycosaminoglycans and C3b, thus exerting decay-accelerating activity and cofactor activity on host cell surfaces. A splice variant product of CFH gene, FHL-1, carries the first seven SCRs of FH and has complement regulatory activity in fluid phase and on the matrix of certain tissues, but it lacks the C-terminal recognition domains and is not capable to bind sialic acids on cell surfaces (3).

Genetic and acquired abnormalities causing FH deficiency or defective activity lead to an uncontrolled alternative pathway activation in the kidney and on blood cells and to secondary C3 deficiency (4). Such abnormalities are associated with different human pathologies which include kidney diseases like the atypical form of the hemolytic uremic syndrome (aHUS) (5), the membrano proliferative glomerulonephritis (MPGN) (6), and the C3 glomerulopathy, and the most common eye disease in adults, age-related macular degeneration (AMD) (7).

Before the introduction of complement-inhibitory drugs, plasma supplementation or plasma exchange was the only available treatment for FH defects, particularly in patients with aHUS and in a few cases with MPGN. With such a therapy, deficient complement regulators are supplemented (8). Plasma exchange therapy in addition removes mutant complement factors and/or autoantibodies directed against complement factors. However, the efficiency of plasma therapy in aHUS depends on the underlying genetic defect (9) and prospective clinical studies are lacking. Data on outcomes with plasma therapy in MPGN and C3G are scanty and limited to case reports (10). In addition, plasma therapy has several limitations. Some patients develop anaphylactic reactions to fresh frozen plasma, which may require cessation of any form of plasma therapy (11).

Plasma perfusions are repeated at regular intervals from twice a week to twice a month, each perfusion lasting 2–3 hours. This treatment is therefore long and recurrent for the patient. The amounts of transfused frozen fresh plasma are significant, which increases the standard risks of frozen fresh plasma perfusion.

The therapeutic potential of purified human FH for MPGN/C3G has been demonstrated with success by *in vivo* treatment in experimental mouse models of FH deficiency. Complement activity was regulated in this C3G model by different FH constructs, including the FH1–5 domains linked to either non-targeting mouse IgG or to anti-mouse properdin antibody (12) and homodimeric mini-FH constructs (13). Treatment with human complement factor H rapidly reverses renal complement deposition in factor H-deficient mice (12). Other recent studies have tested purified human FH for use in a mouse model of bacterial meningitis (13), or are exploring the production of recombinant FH for therapeutic use (14).

**Figure 1:**
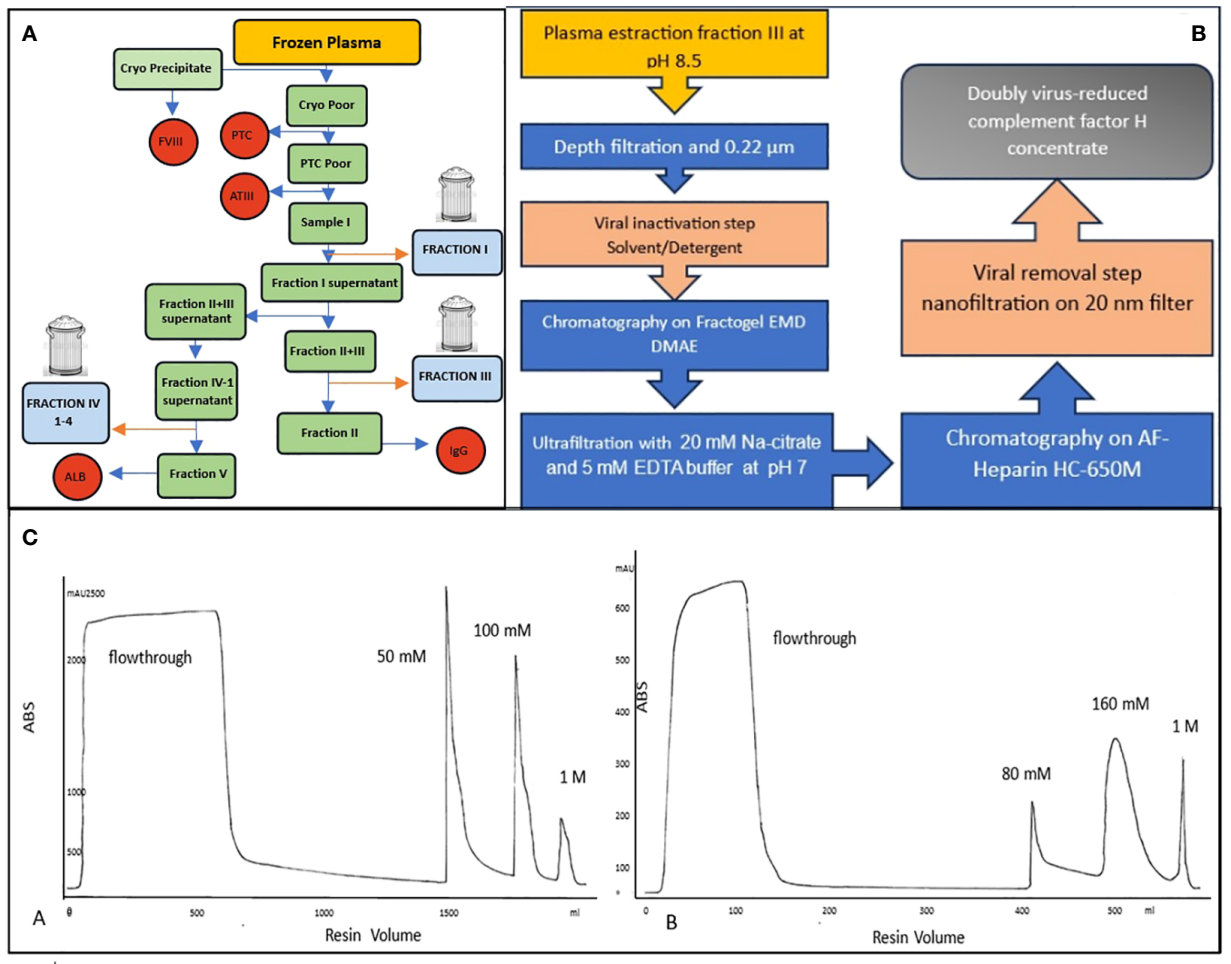
**(A)** Typical Cohn fractionation with 3 waste fractionations: fraction I, fraction III and fraction IV. **(B)** Flow chart of a new purification of human plasma FH, **(C)** Two chromatographic runs: Anionic exchange A=Fractogel EMD DMAE, the most of contaminants are removed in the flowthrough while FH is eluted at 50 mM NaCl. Affinity resin B=AF-Heparin HC 650M was used to further purify FH which elutes at 160mM NaCl after removing the rest of the contaminants in a washing step with 50 mM NaCl.

Therefore, for targeted treatment for diseases requiring FH, it would be desirable for a concentrate to be available; this would facilitate rapid attainment of therapeutic plasma levels with a low protein load. At present, there is no approved FH concentrate available for clinical use. Several groups developed methods to obtain a purified concentrated FH sample from human, rat, or mouse plasma mainly for research purposes (15–18). Another group used the Cohn fractionation method (19) to purify FH from a non-identified fraction of pooled plasma (20), pointing out the necessity to develop a scalable production procedure to obtain a high pure and pathogen-safe FH concentrate. This method is based on the alteration of protein solubility by modifying pH levels, temperatures, and ethanol concentrations. The process separates plasma proteins into five major fractions (**Figure 1A**):

1. Fraction I: Contains as much fibrinogen as possible.
2. Fraction II: Primarily consists of γ-globulins.
3. Fraction III: Composed of lipid-globulins.
4. Fraction IV: Contains α-globulins.
5. Fraction V: Comprises albumins.

The plasma involved in this procedure is typically derived from human blood donations. This blood is collected from numerous donors, and the desired plasma proteins are extracted from these plasma pools through a process known as fractionation. This method has been instrumental in isolating specific proteins for various applications, particularly during wartime, when the demand for certain plasma proteins was very high. It’s a testament to the innovative approaches in protein separation techniques. The Cohn method is still widely used in the industry for the purification of plasma derivatives. However, some fractions are not utilized and are discarded, including Fraction I, Fraction III, and Fraction IV (1–4). Human plasma is a precious resource and represents a very significant proportion of the cost of a plasma-derived product. Production of a new plasma therapy may limit the availability of plasma to afford existing products. The high cost of goods together with the small patient populations can make plasma therapies for rare diseases very expensive, and uneconomic.

This study aimed to identify and purify FH from a waste plasma fraction rather than from whole plasma, allowing more efficient and ethical use of blood and plasma donations. The integrity and function of FH were monitored during all the production steps by *in vitro* assay to evaluate the best purification strategy. Finally, the activity of purified FH was characterized both *in vitro* and *in vivo* in an animal model of C3G to provide the proof of concept that the new purification approach provides a fully active protein.

## Materials and methods

### Materials

FH antigen determination was conducted by commercial ELISA kit (HK342; Hycult Biotechnology Inc., Uden, The Netherlands). Protein concentration was determined by the Bradford Assay using a Coomassie reagent (Blue Stain Reagent, 24592; Pierce, Rockford, IL, USA). Ultradiafiltration (UDF) was performed on membranes with a 100-kDa exclusion limit (Pellicon Mini – Biomax Hydrophilic Polyethersulfone Membrane A Screen; Millipore, Billerica, MA, USA). Complement proteins, control FH, Factor I (FI), Factor B (FB), Factor D (FD) and C3b were from Merck (Merck Millipore, Billerica, MA, USA). The antibody we used for C3 staining is a polyclonal goat IgG fraction to mouse complement C3 that was obtained by immunization with the whole mouse C3. The IgG fraction is prepared from the specific goat antiserum. Thus, the antibody is expected to recognize both inactive C3 and activated C3 fragments. Chemicals were purchased from Merck and used without further purification unless stated otherwise. As far as radiolabeling of FH for subsequent *in vivo* biodistribution study is concerned, ^18^F-fluoride was obtained from a PETrace 860 cyclotron (GE Healthcare, Uppsala, Sweden) irradiating ^18^O-enriched water in silver body target. Radio-TLC was run on silica gel plates (S60-F254, Merck) and radioactive spots were detected using Cyclone Plus phosphor reader (Perkin Elmer). HPLC was used to assess the purity of precursors and intermediate chemicals (Waters 1100 HPLC interfaced to a diode array UV-Vis and a Raytest GINA radio detector). Microfluidic reactions were conducted using an Advion NanoTek system; the system was controlled by version 1.4 of NanoTek software and the plumbing set-up was analog as reported in previous literature (21). The optimization reactions for the fluoroalkynes were run using the software in Automatic Discovery mode.

### FH identification in plasma fractionations and purification

All waste fractions (frz I; frz III and frz IV-1–4) from industrial plasma fractionation (pooled human plasma) were analyzed by ELISA to identify the highest FH content. Thirty-five grams of the waste fraction III, which contains adjuvants (celite and perlite) were dissolved in six buffer volumes (50 mM Tris, 5 mM EDTA, and 300 KIU/ml aprotinin at pH 8.5) for a contact time of at least 30 minutes at room temperature. The soluble fraction III was filtered (3 µm) to remove celite and perlite, S/D-treated by the addition of 1% (w/v) Tween80 and 0.325% (w/v) Tri-N-Butyl-Phosphate and incubated for 30 min at RT. Subsequently, was subjected to weak Ion-exchange (AIX) resin (Fractogel EMD-DMAE, Merck) equilibrated with 50 mM Tris, 5 mM EDTA, 20 mM NaCl pH 8.5 and FH was eluted by the addition of 50 mM NaCl into the equilibration buffer used for the AIX resin. This FH-containing intermediate was adapted for subsequent Heparin affinity resin (AF-Heparin HC 650M; Tosoh Bioscience) by UDF against 20 mM Na-citrate and 5 mM EDTA pH 7. The adapted intermediate was loaded onto the Heparin affinity resin, and FH was eluted with 160 mM NaCl after a washing step with 80 mM NaCl. FH concentrate was finally pre-filtered (0.22 µm), subjected to nanofiltration using filters with pore sizes of 20 nm (Planova 20N; Asahi Kasei) and finally sterile-filtered (0.22 µm).

### SDS-PAGE and Western blot

SDS-PAGE was carried out under reducing and non-reducing conditions using precast 4–12% NuPAGE Bis-Tris protein gels (Thermo, Monza Italy). For each sample, an equal amount of 1 µg was run in the gel. Protein markers were applied (Precision Plus, All Blue pre-stained; Bio-Rad, Hercules, CA, US). Gels were stained with Bio-Safe protein stain (Bio-Rad, Hercules, California, Stati Uniti), and FH purity was compared to purified human FH from commercial sources (341274; Merck).

Immunoblotting was performed by subsequent protein transfer onto nitrocellulose membrane, incubation with anti-FH Goat pAb (341276; Merck) followed by HRP-conjugated goat anti-rabbit-IgG (Merck KGaA, Darmstadt, Germany) or with OX-24 monoclonal antibody (Thermo, Monza, Italy) followed by HRP-conjugated rabbit anti-mouse-IgG (DAKO), washing and monitoring upon substrate exposure with 4Opti-CN-substrate-Kit (Bio-Rad, Hercules, California, Units States) Hercules, California, Stati Uniti) using a digital imaging system Chemidoc (Bio-Rad, Hercules, California, Units States).

### Evaluation of structural integrity

To evaluate the integrity of FH during the production steps, samples were analyzed by SDS-PAGE under reducing conditions, and gels were analyzed by densitometry with TotalLab Quant software (TotalLab Ltd, Newcastle upon Tyne, UK). The integrity was calculated as a percentage of from intensity of the bands corresponding to intact or truncated form over total FH.

### Native-polyacrylamide gel electrophoresis

To investigate the presence of oligomers, samples were separated using native PAGE on 4–15% Mini-PROTEAN TGX Stain-Free Protein gels (Bio-Rad, Hercules, California, Unit States) in Tris/Glycine running buffer for 5 h at 100 V. After run, stain-free gels were exposed to UV light to activate the embedded tri halo compound and make proteins fluorescent, images were acquired with ChemiDoc MP Imaging System (Bio-Rad, Hercules, California, Unit states).

### Quantification of accompanying plasma proteins

C3, C4, immunoglobulin A, E, G and M (IgA; IgE, IgG; IgM), transferrin and fibrinogen were quantified using rabbit polyclonal antibodies against those proteins on a BN100 nephelometer (Siemens, Munich, Germany).

Total protein concentration was assessed by Bradford assay (Pierce, Waltham, Massachusetts, USA), according to the manufacturer’s instruction, using albumin as standard.

### Protein digestion for MS analysis

Samples are desalted using SPE (Solid Phase Extraction, PIERCE). One hundred and fifty µl of Ammonium bicarbonate 50 mM were added to 100 µl of a sample at pH 8.0. Twenty μg of the resulting proteins were further processed. Proteins were thus reduced with 5 mM dithiothreitol at 80°C for 20 min and alkylated for 30 min with 10 mM iodoacetamide at 37°C. Digestion was carried out by incubating overnight at 37°C in a solution containing trypsin (Roche, Germany) at 1:100 ratio with the substrate. The resulting peptide solution was loaded on a C18 cartridge in order to purify peptide solutions and filtered with 0.22 μm filter. Peptides were diluted to 100 μL by 5% ACN/0.1% FA.

### HPLC- MSMS analysis

Chromatographic separation of peptides was performed using a nano-HPLC system (Thermo, Monza, Italy) in duplicates. Samples were loaded through a pre-column cartridge (PepMap-100 C18 5 μm 100 A, 0.1 × 20 mm, Thermo, Monza, Italy) and then resolved in a C18 PepMap-100 column (3 μm, 75 μm × 250 mm, Thermo, Monza, Italy) at a flow rate of 300 nL min^−1^. Runs were performed with eluent A H_2_O (0.1% HCOOH), eluent B: 80% acetonitrile (0.1% HCOOH) 20% H_2_O (0.1% HCOOH), starting with 5% of B and increasing to 35% in 40 min, then up to 100% in 1 min and isocratic for 8 min then back to the initial composition and equilibrating for 10 min.

The detection was done by an Orbitrap Q Exactive Plus high resolution mass spectrometer operated in positive ion mode. The operating parameters were optimized using standard proteins digested in the same condition as for the human samples and were as follows: spray voltage 1.9 kV, Max Spray Current:50.00 Probe Heater Temp.350°C, S-Lens RF Level:50.00, auxillary gas flow 5 arbs. Data were acquired in centroid mode across the 400–1500 m/z range. A false discovery rate (FDR) analysis was accomplished by using the integrated tools in Proteome Discover software with a confidence level of 95%. The identification of proteins are related parameters were obtained using MASCOT and Sequest-HT search engine. The mass spectrometry proteomics data have been deposited to the ProteomeXchange Consortium via the PRIDE partner repository (22) with the dataset identifier PXD050268.

### Cofactor activity

The fluid-phase FH cofactor activity for cleavage of C3b by FI was assessed using purified C3b and CFI (Merck). This test was used both to monitor production steps and drive the purification procedure and on the final FH concentrate.

To test FH activity from purification intermediates containing other proteins it was necessary to further purify FH by a one-step affinity chromatography on a FH-specific antibody (5H5) with an already described method (23). Affinity-purified FH was then used to compare different purification intermediates. In this case we used as control a FH directly purified from plasma with the same method. On the contrary, when the test was performed on the final FH concentrate, this was used directly in the assay without any further purification.

Briefly, 500 ng of C3b and 125 ng of CFI were mixed with varying amounts of FH in a 20-µl reaction with PBS buffer. For affinity-purified FH from different purification intermediates, limiting amounts were used (0.5 - 1 - 2.5 ng) while for the final FH concentrate a wider range between 0.25 and 200 ng was used.

Reactions were incubated for 30 min at 37°C and stopped by the addition of reducing SDS sample buffer. Samples were analyzed by SDS-PAGE under reducing conditions followed by Coomassie staining. The cofactor activity was calculated as a decrease in the ratio between α’ chain of C3b over β chain.

### C3b binding assay (ELISA)

The assay was modified from a previous study (24). Microtiter plates were coated with 250 ng of C3b (Merck) in coating buffer (0.05 M carbonate-bicarbonate, pH 9.6) overnight at 4°C. After washing, plates were blocked with 3% BSA for 1 h at room temperature. Wells were incubated with purified or commercial FH (Merck) from 800 to 12.5 ng for 1 h at 37°C. Binding was detected with chicken anti-FH pAb (in-house) (23) and HRP-conjugated anti-chicken IgY (G135A - Promega, Fitchburg, WI, USA) followed by TMB (KPL, Gaithersburg, MD, USA) development. After stopping with 2 M H2SO4 the absorbance was measured at 450 nm on a Spectra Max 190 photometer (Molecular Devices, Eugene, OR).

### Decay acceleration activity

The ability of FH to dissociate Bb fragments from C3 convertase complexes (C3bBb) was assessed by ELISA. Briefly, microtiter plates were coated with 250 ng of C3b (Merck) in coating buffer (0.05 M carbonate-bicarbonate, pH 9.6) overnight at 4°C. To allow the formation of C3 convertase complexes, 400 ng of FB, 30 ng of FD (Merck) and 1.5 mM NiCl2 in 10 mM phosphate buffer pH 7.2 supplemented with 25 mM NaCl and 4% BSA were added, and the plate was incubated for 2 h at 37°C. After washing, increasing concentrations of FH (from 2 µg to 0.1 ng) were added and incubated for 45 min at 37°C to allow displacement Bb fragment. The remaining C3 convertase complexes were detected by goat polyclonal antibody to human FB (341272; Merck) followed by rabbit anti-goat IgG-HRP (401515**;** Merck). The signal was revealed with ***O*-**phenylenediamine (OPD), stopped with 2 M H2SO4 and the absorbance was read at 492 nm.

### Radiolabeling of FH for *in vivo* biodistribution by PET imaging

In order to assess the kinetics of the purified FH *in vivo* quantitatively, a radiosynthesis procedure was devised and implemented, involving a microfluidic approach. Fluorine-18 (^18^F, decay half-life T1/2 = 109.7 min) was selected as the radionuclide, due to favorable radiochemistry and widespread use in Positron Emission Tomography (PET) imaging. Chemicals used were purchased from Merck (Milano (MI), Italy), unless stated otherwise.

### (N3-PEG)@FH precursor synthesis

One hundred twenty-five µg of human FH concentrate was dissolved in 500 µL of PBS 50 mM at pH 6.5 and stirred at room temperature for 10 minutes. In a second vial, 2.9 mg of N3-PEG (4)-COOH (IRIS Biotech GmbH, Marktredwitz, Germany) was activated using a solution of 1.1 mg of N-Hydroxysuccinimide (NHS) and 1.5 mg of (1-ethyl-3-(3-dimethylaminopropyl) carbodiimide hydrochloride) (EDC, Fluka, Brescia, Italy) in 200 µL of 2-(N-morpholino) ethanesulfonic acid (MES, Thermo, Monza, Italy) at pH 4.5.

The activation reaction was left standing at room temperature for 20 minutes. After this time, the solutions from both vials were combined and the resulting mixture was basified by 25 µL of N,N-Diisopropylethylamine (DIPEA) to a final pH of 8.5. The reaction was kept at room temperature for 2 hours without agitation. The final product was separated from an excess of small molecular weight reagents on a Sephadex G-25 column using a 0.25 mM NaCl aqueous solution as eluent. The (N3-PEG)@FH was further purified by ultracentrifugation, suspended in water, lyophilized, and the resulting powder was analyzed by HPLC.

### Microfluidic optimization of tosyl-alkyne radiolabeling

Two compounds, 5-tosyloxy-1-pentyne (PeTos) and 6-tosyloxy-1-hexyne (HeTos) were synthesized from the corresponding alcohols (25) and used in the optimization process. The system tests different temperatures, solvents, and ratios of precursor to labeling solution. The reaction mixtures were analyzed and pure [^18^F]fluoroalkynes were obtained by heating and condensing the gaseous products. The process was efficient and precise, allowing for the production of radiochemically pure compounds.

### Microfluidic synthesis of [^18^F]fluoropentyne ([^18^F]FPe) for CuAAC reaction

The [^18^F]FPe was synthesized from the corresponding tosylate using a microfluidic system under optimal radiolabeling conditions. The starting [^18^F]fluoride was captured on an ion exchange column and eluted with a solution of Kryptofix 222 (K_222_) in acetonitrile (21). The radioactive solution and PeTos precursor solution were delivered into a microfluidic reactor, heated at 130°C, to obtain the final product in high radiochemical purity and 92 ± 3% RadioChemical Yield (RCY) (26, 27). The final product was then distilled into a second vial containing a (N3-PEG)@CFH solution, leaving unreacted fluoride and reagents in the first vial. The procedure allowed obtaining 15 ± 10MBq of [^18^F]FPe 45 min after the start of the synthesis.

### Synthesis of [^18^F]FPe-(trN-PEG)@FH

[^18^F]FPe was collected in a vial containing a solution of (N3-PEG)@FH in water. After distillation, the mixture was stirred at 45°C for 5 min. A solution of CuI and Tris(benzyltriazolylmethyl)amine (TBTA) in THF/H_2_O was added to the reaction and stirred at 45°C for an additional 10 min. The solution residue was diluted with isotonic saline containing 1% ethanol and purified by gel filtration. The quality of [^18^F]FPe-(trN-PEG)@FH was assessed using a HPLC interfaced to a diode array UV-Vis and a GINA radio detector.

### PET/CT image acquisition and analysis

The images were acquired using an IRIS CT/PET instrument designed for high-resolution imaging of small animals (27, 28). This instrument allowed for 3D and time-resolved quantification of tracer kinetics in the entire mouse body.

All imaging procedures were performed under general anesthesia in accordance with international guidelines and Italian laws for the care and use of laboratory animals, demanded by the European Directive (Directive 86/609/EEC of 1986 and Directive 2010/63/UE) and Italian laws (D.lgs. 26/2014). Each animal was anesthetized with isoflurane and oxygen, and positioned on the imaging bed once the correct degree of anesthesia is reached (29).

### Imaging protocol

[^18^F]FPe-(trN-PEG)@FH was formulated in saline before injection, and used for *in vivo* PET imaging of both control and Cfh-/- mice. The tracer was intravenously administered to two distinct groups of mice: one consisting of C57BL6 mice and the other composed of Cfh-/- mice. The dynamic acquisition of the tracer was carried out from 5 min to 160 min post-injection. The image reconstruction was done using 3D ordered subset expectation-maximization (OSEM), with corrections for radioactive decay, random coincidences, and dead time. Low-dose micro-CT imaging was also performed after PET on the same integrated scanner. Both PET and CT images were exported in standard DICOM format after reconstruction for further image processing/quantification.

### Quantitative image analysis

Analysis of PET/CT images was performed using AMIDE software v 1.0.4 three-dimensional regions of interest (ROIs) were traced on the left ventricle cavity, whole brain, liver, kidneys, gallbladder and bladder. Standardized Uptake Value (SUV) was calculated on each ROI and on each time frame, and the results were plotted against time for each animal. On each of the two groups (CTRL and Cfh-/-), average values, range, median and quartiles of organ uptake at each time point were calculated.

### Animal models experimental protocols

These studies were done in the mouse model of C3 glomerulopathy in Cfh−/− mice. Comparing Cfh−/− with wild-type mice, Cfh−/− mice developed severe albuminuria starting at 8 months of age.

#### Study 1: single administration

Cfh−/− mice (n=4, 2–4 months old) received a single intraperitoneal injection (0.5 mg in 200 microliter PBS) of human plasma FH concentrate. Four Cfh−/− mice received PBS as controls. At baseline (before injection), and at 2 and 6 h after injection we evaluated serum C3 levels by ELISA. At 24 hours after injection, the animals were sacrificed. Blood was collected for the evaluation of serum C3 levels and human FH levels by ELISA. The kidneys were collected for immunofluorescence analysis of C3 and C5b-9 deposition in glomerular capillaries and tubuli. Sections of kidney, liver, eye, and heart tissues were stored for future analysis by light microscopy and ultrastructural analysis by transmission electron microscope.

#### Study 2: multiple administrations

Four groups of Cfh−/− mice (n=4 each, 2–4 months old) have been studied as follows:

Group 1: Cfh−/− mice received two daily intraperitoneal injections (0.5 mg in 200 microliters PBS) of human plasma FH concentrate. At 48 hours (24 hours after the second injection) the animals were sacrificed. Group 2: Cfh−/− mice received three daily intraperitoneal injections (0.5 mg in 200 microliters PBS) of human plasma FH concentrate. At 72 hours (24 hours after the third injection) the animals have been sacrificed. Group 3: Cfh−/− mice received four daily intraperitoneal injections (0.5 mg in 200 microliters PBS) of human plasma FH concentrate. At 96 hours (24 hours after the fourth injection) the animals were sacrificed. Group 4: Cfh−/− mice received five daily intraperitoneal injections (0.5 mg in 200 microliters PBS) of human plasma FH concentrate. At 120 hours (24 hours after the fifth injection) the animals were sacrificed. Serum C3 levels were evaluated in all animals at baseline. At sacrifice, blood was collected for the evaluation of serum C3 levels and human FH levels. Human FH levels were also measured at baseline in 4 Cfh−/− mice as negative controls of the assay. The kidneys were collected for immunofluorescence analysis of C3 and C5b-9 deposition in glomerular capillaries and tubuli. Three wild-type C57/BL6 mice were also studied as controls.

### Immunofluorescence analysis of renal tissue

Three-μm OCT-fixed frozen sections were used for the evaluation of C3 and C5b-9 staining. For C3 deposition, sections were incubated with FITC-conjugated goat anti-mouse C3 Ab (1:200; Cappel) and with Rhodamine-labeled Lens Culinaris Agglutinin (1:400) and DAPI to label kidney structures and cell nuclei, respectively. For C9 staining, rabbit anti-mouse C9 (1:1600) and Cy3-conjugated goat anti-rabbit secondary Abs (1:300) were used. Fluorescein-labeled Lens Culinaris Agglutinin (1:400) and DAPI were used to label kidney structures and cell nuclei, respectively. Isotype-matched irrelevant Abs were used as negative controls. Glomerular C3 and C9 staining was scored from 0 to 3 (0, no staining or traces (<5%); 1, staining in <25% of the glomerular tuft; 2, staining affecting 26 to 50%; 3, staining >50%). Tubular C3 and C9 staining were scored from 0 (no staining) to 1 (granular staining), 2 (linear interrupted staining), to 3 (linear staining).

### Statistical analysis

*In vivo* animal model data analysis were reported as mean ± SD and were analyzed by one-way ANOVA (MedCal software). Values of p < 0.05 were considered statistically significant. For PET biodistribution data, two-tailed Welch’s t-test was carried out for comparison of the organ’s SUV data at each time point, between the control and diseased (Cfh-/-) group.

## Results

### FH process purification and characterization

Based on Cohn fractionation, the plasma proteins were separated with different ethanol concentrations and pH levels into five major fractions (19) as much as possible fibrinogen in Fraction I, y-globulins in Fraction II, lipid-globulins in Fraction III, α-globulins in Fraction IV, and albumins in Fraction V. Proteins extracted and purified from plasma have a wide range of therapeutic uses, including treatment for immune deficiencies, neurological diseases, autoimmune disorders, and bleeding disorders, among others (30). However, the demand for specific proteins can vary, and this, along with other factors as the difficulty of further purification, can lead to certain fractions being discarded (Fraction I; III and IV (1–4)). To identify in which waste fraction FH was present we analyzed the proteome of the principal unused fractionation intermediates from an industrial plasma fractionation plant. FH was found in Cohn Fraction III and its presence was confirmed by ELISA. Cohn Fraction III contains a very high concentration of FH with a recovery of 70% compared to cryo-poor plasma (**Figure 1A**) addressing its use as a starting material for FH purification.

Two scalable chromatography steps were applied to purify a new FH concentrate resulting in 87.96% purity (**Figure 1B**).

The novel process developed for the purification of human plasma FH is schematized in **Figure 1C** and comprises two chromatographic separations, including pathogen elimination and inactivation by Solvent – Detergent (S /D) treatment and nanofiltration.

Each step of the purification procedure was monitored for FH contents, integrity, and functionality, to choose the purification protocol that best preserves FH integrity and activity.

In the process optimization study, aprotinin was added as a protease inhibitor only in the phase of extraction of FH from fraction III. This allowed for a reduction of the percentage of degraded protein, (**Supplementary Material Table S1**), resulting from the analysis in SDS-PAGE (data not shown) and subsequent densitometry of the purified product obtained from samples extracted with or without aprotinin (300 KIU/ml).

The purification intermediates were analyzed by SDS-PAGE and western blot: the electrophoresis was performed in both reducing and non-reducing conditions (**Supplementary Material Figure S1**). The analysis reveals the presence of FH in all the intermediates, the bands corresponding to cleaved form of FH (130 and 35 KDa) are also evident upon reduction. A faint band around 35 KDa is also present in non-reducing conditions only in the filtration intermediate, probably corresponding to the splice variant FHL-1, however this band is no longer visible in subsequent purifications.

**Figure 2:**
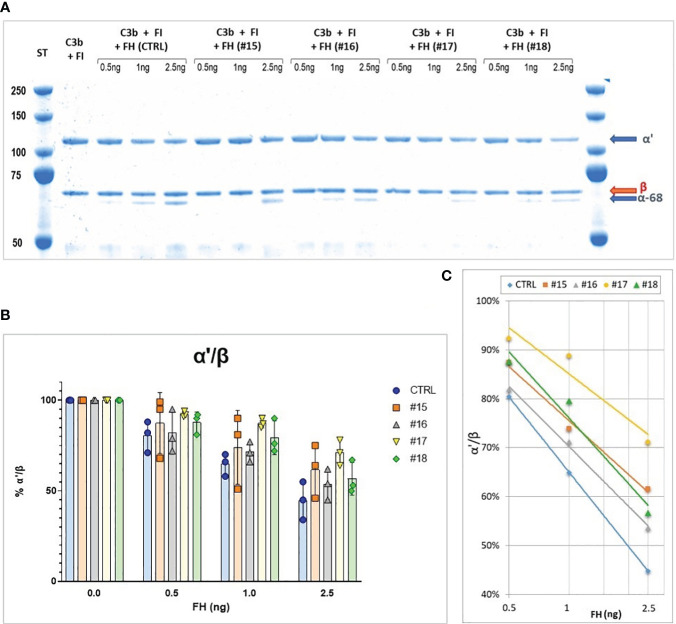
**(A)** SDS-PAGE and Coomassie staining of a Cofactor assay on 4 different purification intermediates (#15, #16, #17, #18) and a Control (CTRL). The samples were subjected to affinity purification with a FH-specific antibody to exclude contaminating protein before performing the test, the control was purified directly from human serum by the same affinity chromatography. **(B)** α’/β chains ratio obtained for the different samples, means and SD from three independent assays are indicated. **(C)**, Curves generated by linear-log regression from mean results.

FH obtained from different purification intermediates was analyzed by cofactor assay to monitor FH activity. Different samples were directly compared by plotting α’-chain/β chain ratio and generating a curve by linear-log regression. **Figure 2** reports an example of an analysis on 4 different samples obtained during the development of the purification protocol. Two batches (#16 and #18) were produced using aprotinin in the phase of extraction of FH from fraction III and show a better activity, confirming the importance of adding this inhibitor during purification. Moreover, batch #16, which, unlike batch #18, was stored in a buffer containing glycine as a stabilizer, preserves the best activity.

### Characterization of the final product

Upon completion of the process, the overall yield of the FH process purification was found to be 38.60%. The purified FH demonstrated a purity level of 92%, which is represented as the ratio of FH antigen to the total protein, with an antigen concentration of 3.093 mg/ml. Our process increases the purity by 9.1 fold compared to Cohn fraction III (starting material) and 211.6-fold over FH present in plasma (**Table 1**).

**Table 1:** Step Recovery and purities of complement Factor H (FH): The FH amounts are obtained by ELISA as described in Material and Methods.

FH-rich intermediates	FH amount (mg/l)	Total protein (mg/ml)	FH Purity (%)	FH Step process recovery (%)	FH process recovery (%)
Human plasma	414.00	80.00	0.52	–	–
Plasma fraction (starting material)	1000.74	7.72	12.96	–	–
Miniprofile filtration	541.34	5.55	9.74	95.03	95.03
Anion-exchange chromatography eluate	675.02	1.76	38.30	71.83	66.21
Heparin affinity chromatography eluate	1167.95	1.32	87.96	71.09	43.38
Purified FH (20N + 0.22 µm)	3093.93	2.81	92.07	100.00	38.60

The purity % is a ratio between the amount FH and total protein.

The purified FH was concentrated about 7 times, by a 30 KDa cut-off centrifugal concentrator Vivaspin, and tested for C3, C4, Immunoglobulin (IgA, IgM, IgE, IgG), fibrinogen, albumin, and transferrin by nephelometric analysis.

Among the analyzed proteins of three different process, complement C3 protein has the highest concentration with 255.5 ± 10.6 mg/L and Immunoglobulin A and M with respectively 12.85 ± 0.49 mg/L and 13.1 ± 0.14 mg/L (**Table 2**).

**Table 2:** Nephelometric analysis of the most important contaminants.

Contaminant	Concentration (mg/ml)	Limit of Detection (mg/ml)
Factor H	2.9 ± 0.12	
Complement component C3	0.26 ± 0.01	0.0084
Complement component C4	< DL	0.0032
Immunoglobulins G	0.0044 ± 0.0004	0.0035
Immunoglobulins A	0.0128 ± 0.004	0.0035
Immunoglobulins M	0.0131 ± 0.001	0.0035
Immunoglobulins E1	< DL	0.0035
Immunoglobulins E2	< DL	0.0035
Transferrin	< DL	0.0174
Albumin	< DL	0.0177
A2-macroglobin	< DL	0.0086
Fibrinogen	< DL	0.0266
Fibronectin	< DL	0.0417

C3, IgA and IgM, are the most representative contaminant protein.

The results are confirmed by proteomic analysis, where different batch samples of FH (N=3, detailed results are reported in **Supplementary Material Table S2**) were processed and analyzed using the protocol reported in the “methods” section The mean identified proteins were 185 ± 18 (Mean ± SD) with a statistical significance of ≥ 95%. In **Supplementary Material Table S3** are reported the common proteins comparing the three batches.

FH coverage was 71 ± 1,5% (Mean ± SD) by peptide analysis using Sequest HT search engine. Data are available via ProteomeXchange with identifier PXD050268.

### SDS-PAGE and Western blot

**Figure 3:**
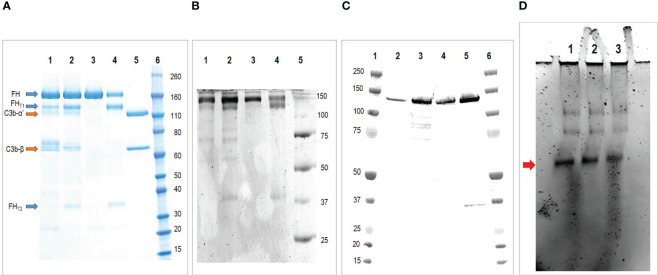
Characterization of the purified FH. **(A)** SDS-PAGE under reducing condition of purified FH. Lane 1 and 2 two batches of purified FH; Lane 3, commercial FH; Lane 4, commercial FH cleaved with trypsin; Lane 5, C3b; Lane 6, molecular weight standard. The analysis reveals the presence of FH integral form (apparent MW 170 kda) together with the larger (130 kda) and smaller (35 kda) fragments of the truncated form. Moreover it reveals the presence of C3b as major contaminant. **(B)** Western blot under reducing condition of purified FH. Lane 1 and 2 two batches of purified FH; Lane 3, commercial FH; Lane 4, commercial FH cleaved with trypsin; Lane 5, molecular weight standard. This analysis confirms the presence of FH integral form (170 kda) together with the larger (130 kda) and smaller (35 kda) fragments of the truncated form. **(C)** Western blot under non reducing condition of purified FH with antibody OX-24. Lane 1, molecular weight standard; Lane 2 and 3 two batches of purified FH; Lane 4, commercial FH; Lane 5, human serum (used as positive control); Lane 6, molecular weight standard. This analysis exclude the presence of FHL-1 in our preparations. **(D)** Native-PAGE analyses of purified FH. Lane 1, purified FH stored at 1.5 mg/ml; Lane 2, purified FH stored at 3 mg/ml; Lane 3, commercial FH stored at 1 mg/ml. The major form indicated by the arrow corresponds to the monomer, the higher molecular-weight bands denote oligomeric forms.

The final FH concentrate was analyzed by SDS-PAGE under reducing condition (**Figure 3A**), the analysis revealed the presence of the cleaved form of FH and, as already observed with other methods, the presence of C3b as the major contaminant. The presence of the cleaved form of FH was also shown by Western Blot analysis under reducing condition with a polyclonal antibody against FH (**Figure 3B**). Finally, to exclude the presence of the FHL-1 splice variant, which could be confused with the lower band of the cleaved form, a non-reducing Western blot with the OX-24 antibody was performed (**Figure 3C**). This antibody recognizes an epitope in the SCR-5 of FH and detects also FHL-1. Under non-reducing condition the cleaved form of FH is held together by a disulfide bridge and migrates as the integral form, thus the only form visible around 35 KDa is FHL-1. This analysis excluded the presence of FHL-1 which is visible in a sample of human serum used as a control, but not in our concentrates.

### Native-polyacrylamide gel electrophoresis

It is hypothesized that FH oligomerizes when stored at high concentrations. To evaluate the presence of high molecular weight species, we conducted a Native PAGE analysis. We examined two batches of our purified FH, with concentrations of 1.5 mg/ml and 3 mg/ml respectively, along with a commercially available purified FH preparation of 1 mg/ml (as shown in **Figure 3D**). The results were consistent across all samples, displaying a prominent band corresponding to the monomer and fainter bands of higher molecular weight, which correspond to the oligomeric forms.

### Functional analysis of purified FH

The activity of our purified FH preparation was tested in three different functional assays.

#### Cofactor assay

**Figure 4:**
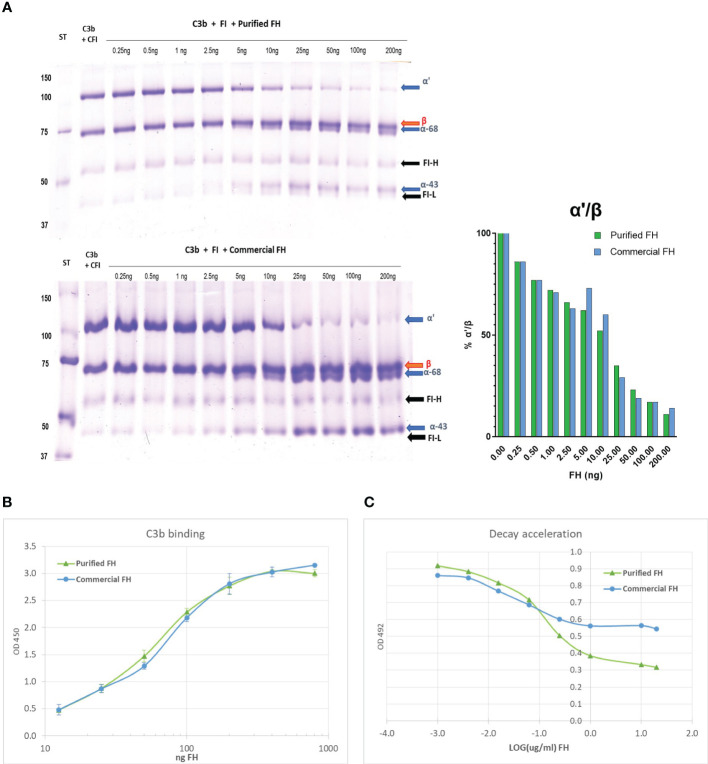
*In vitro* activity of the purified FH compared with a commercial FH (Merck). **(A)** Cofactor assay: C3b and FI were incubated with different amounts of purified FH, run on a reducing SDS-PAGE and stained with Coomassie. In the presence of FH, FI cleaves C3b and the degradation products iC3b-α68 and iC3b-α43 are visible. The bands corresponding to the heavy (FI-H) and light (FI-I) chain of FI are also evident in the gel. **(B)** C3b binding assay: Purified FH can bind surface-bound C3b in a dose-dependent manner, similar to commercial FH. The binding of FH to immobilized C3b was visualized in an ELISA assay by the use of an antibody to human FH. **(C)** Decay acceleration activity: Purified FH displays decay acceleration activity on C3 convertase complexes. The decay of C3bBb was enhanced by the presence of an increasing concentration of FH. The decrease of bound Bb to the intact complex was visualized by the use of an antibody to human FB. Results are presented as LOG of FH concentration.

FH acts as a cofactor for FI-mediated cleavage of C3b to iC3b in the fluid phase. The cleavage products of C3b α’-chain, α68, and α43 iC3b chains can be visualized on a reducing SDS-PAGE. Purified FH displays cofactor activity, a minimum of 2.5 ng is sufficient to induce C3b cleavage, as shown by the reduction of α’-chain and the presence of α68 and α43 chains (**Figure 4A**). Results were comparable to those obtained with commercial FH.

#### C3b binding

The ability of purified FH to bind surface-bound C3b was assessed by ELISA assay. We observed a dose-dependent binding of purified FH to immobilized C3b, comparable results were obtained with the commercial FH (**Figure 4B**).

#### Decay acceleration activity

Purified FH displays a dose-dependent decay acceleration activity on C3 convertase complexes. Increasing the amount of FH enhanced the displacement of the Bb fragment causing a decrease in the intact C3 convertase (C3bBb). The effect was comparable to that obtained with purified FH from a commercial source (**Figure 4C**).

#### Radiochemistry

As conjugation partners, [^18^F]fluoropentyne and [^18^F]fluorohexyne were radiolabeled using a microfluidic flow approach from respective tosylate precursors, achieving maximum radiochemical conversion (RCC) of 96 ± 9% and 81.5 ± 5% respectively. Therefore, [^18^F]fluoropentyne was chosen and simple distillation was effective in recovering such prosthetic group in high purity, and reacted quantitatively with the azide-functionalized FCH.

[^18^F]FPe-(trN-PEG)@FH was produced via a 2-step process, comprising the radio fluorination of the 5-tosyl-pent-1-yne under microfluidic conditions (31) (92% radiochemical yield, RCY), its purification by distillation, and a quantitative click reaction conjugation with an aqueous solution of (N_3_-PEG)@FH and CuCl/TBTA in 10min at 45°C. The process led to the desired product in >95% radiochemical purity (RCP).

### Animal experiments

#### *In vivo* PET imaging

**Figure 5:**
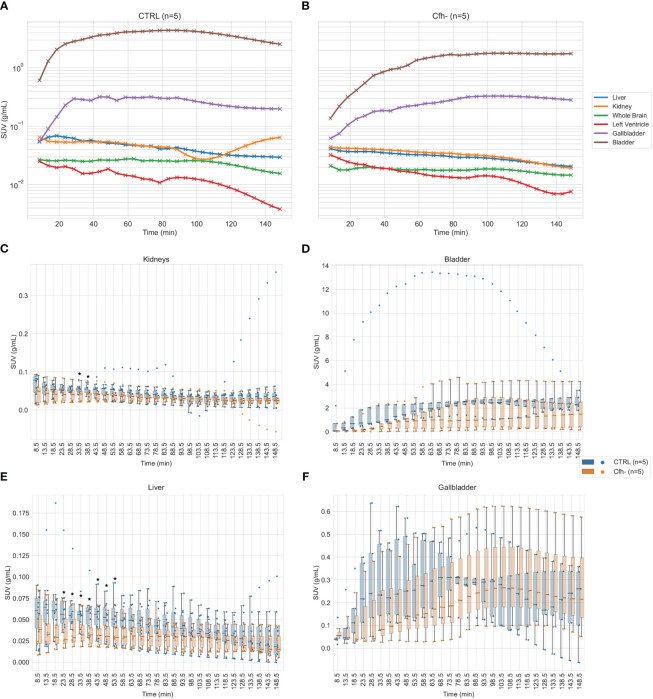
**(A, B)**: Average tracer kinetics of the ^18^F-labelled FH in organs, for the control group (left) and Cfh-/- group (right). Activity levels are expressed in terms of Standardized Uptake Values (SUV). **(C–F):** Organ-specific distribution of tracer uptake for kidneys (both sides) **(C)**, bladder **(D)**, liver (all ROIs) **(E)** and gallbladder **(F)**. Individual data points for each subject at each time are superimposed to boxplots for better understanding of the intragroup and intergroup variability. Statistical significance (p<0.05) has been marked with asterisks at the corresponding time frames.

Previous works have reported the tagging and imaging of FH analogues, using I-125 labelling (32) Tc-99m (33) or optical tags (34), however, these studies presents limitations in terms of imaging performance (i.e. planar scintigraphy or autoradiography), scope (i.e. identification of myocardial damage) and pharmacological assessment (i.e. using FH fragments or recombinant complement). We therefore decided to design an imaging tracer based on entire FH that can be used in PET imaging, thus improving on these aspects. The biodistribution of the labeled product imaging was assessed in CTRL and Cfh-/- mice by dynamic PET. **Figure 5** shows the average tracer kinetics in the selected organs, for the two groups. The reported activities are expressed as Standardized Uptake Values (SUVs) and take into account the different weights of each animal. Indeed, control mice were heavier than Cfh-/- countermates (CTRL: 42.6 ± 3.0 g; Cfh-/-: 21.7 ± 1.6 g p<0.001). A different shape of activity accumulation in both the bladder and the gallbladder for the Cfh-/- mice during the first 60’ post-injection is apparent in the observed curves.

**Figure 6:**
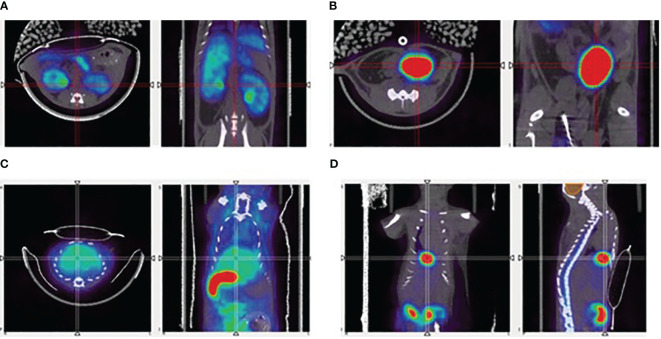
Representative PET/CT images of the ^18^F-labelled FH, in mice, showing uptake in **(A)** kidneys, **(B)** bladder, **(C)** liver, and **(D)** gallbladder.

The renal curves, inclusive of tracer activity for both cortical (extraction) and medullary (excretion) components, resulted significantly different in the time range of 40 min post-injection (CTRL: 0.055 ± 0.011 g/ml; Cfh-/-: 0.040 ± 0.017 g/ml, p<0.05, at 33.5 min post-injection; CTRL: 0.054 ± 0.012 g/ml; Cfh-/-: 0.040 ± 0.017 g/ml, p<0.05, at 38.5 min post-injection) Also, the uptake in the liver was different in the time range of 20–55 min post-injection (CTRL: 0.065 ± 0.034 g/ml; Cfh-/-: 0.037 ± 0.021 g/ml, p<0.05, at 23.5 min post-injection; CTRL: 0.051 ± 0.022 g/ml; Cfh-/-: 0.033 ± 0.015 g/ml, p<0.05, at 53.5 min post-injection). Intra and inter-group variability of the tracer uptake as a function of time from injection is shown in **Figure 5**. Apart from the gallbladder, Cfh-/- mice show much intragroup variability than control mice, with lower SUV values especially in the bladder and kidney as shown in **Figure 5**. Both bladder and gallbladder curve also show a delayed uptake in Cfh- mice as compared with controls. Two out of 5 Cfh-/- mice did not show any tracer uptake in the gallbladder, explaining the high variability in this group (**Figure 5F**). Some representative PET/CT images of the [^18^F]FPe-(trN-PEG)@CFH are shown in **Figure 6**.

#### Effect of the human FH concentrate on C3 levels in Cfh−/− mice

**Figure 7:**
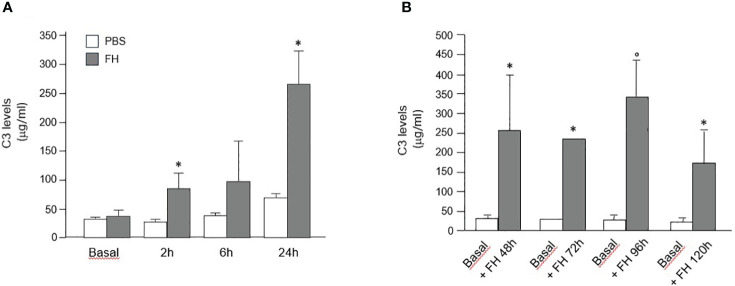
**(A)** Mean+SD values (μg/ml) of plasma C3 levels measured in Cfh−/− mice at baseline and at different time points after a single injection of PBS (control) or human FH concentrate. * P<0.05 versus PBS control group. **(B)** Mean+SD values (μg/ml) of plasma C3 levels were measured in Cfh−/− mice at baseline and at sacrifice (48 h, 72 h, 96 h, and 120 h) after multiple injections of a human FH concentrate. *P<0.05 versus baseline; °P<0.01 versus baseline.

In Cfh−/− mice treated with a single injection of human FH concentrate, plasma C3 levels progressively increased at 2, 6, and 24 h after injection, and values were significantly higher than those recorded in the same Cfh−/− mice at baseline and at the corresponding time points in PBS treated Cfh−/− mice (**Figure 7A**). Similarly, C3 levels at sacrifice were significantly higher than values at baseline in Cfh−/− mice treated with daily injections of human FH concentrate and sacrificed at 48 h, 96 h, and 120 h (**Figure 7B**). In the group sacrificed at 72h, C3 levels increased in 2 out of 4 animals while for the other 2 animals, not enough plasma could be obtained (**Figure 7B**). However, at all-time points, plasma C3 values in Cfh −/− mice treated with the FH concentrate remained significantly lower than C3 values in C57/BL6 wild-type mice (909.6 ± 145.68 μg/ml). These results indicate that administration of a FH concentrate partially but significantly prevented C3 activation and consumption in the circulation of Cfh −/− mice.

#### Evaluation of human FH levels in CFH−/− mice

Human FH levels in plasma of 4 Cfh−/− mice at baseline were undetectable, as expected. At variance, human FH was detectable at all time points after either single or multiple injections of human FH concentrate starting from 32.64 μg/ml at 24 h to 41.01 μg/ml at 96 h concentration of FH (**Supplementary Material Figure S2**).

#### Effect of the human FH concentrate on C3 staining in the kidney of Cfh−/− mice

**Figure 8:**
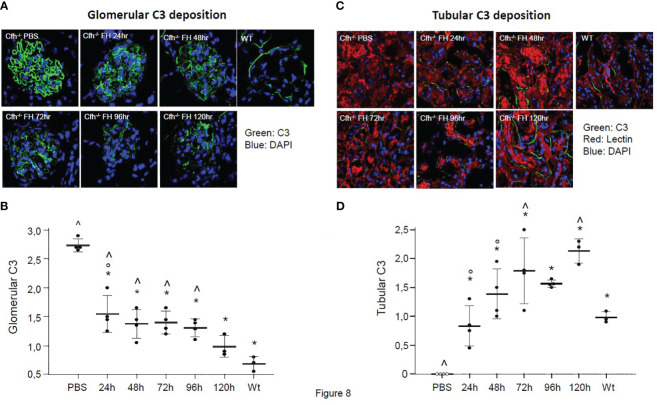
**(A)** Representative images of glomerular C3 staining (in green) in Cfh−/− mice treated with PBS or with the human FH concentrate, and in wild-type (WT) mice. Nuclei are stained by DAPI (in blue). **(B)** Results of semiquantitative evaluation of glomerular C3 staining in Cfh−/− mice treated with PBS or with the human FH concentrate, and in wild-type (WT) mice. Mean ± SD, individual values are represented as black dots. *p<0.05 versus PBS control group; ° p<0.05 versus 120 h group; ^ p<0.05 versus WT group. **(C)** Representative images of tubular C3 staining (in green) in *Cfh* ^−/−^ mice treated with PBS or the human FH concentrate and in wild-type (WT) mice. Nuclei are stained with DAPI (in blue), cell membranes with Rhodamine-labeled Lens Culinaris Agglutinin (in red). **(D)** Results of semiquantitative evaluation of tubular C3 staining in Cfh−/− mice treated with PBS or with the human FH concentrate, and in wild-type (WT) mice. Mean ± SD, individual values are represented as black dots. *p<0.05 versus PBS control group; ° p<0.05 versus 120 h group; ^ p<0.05 versus WT group.

We observed intense staining for C3 in the glomeruli of PBS-treated Cfh−/− mice, which was significantly higher than glomerular C3 staining in wild-type mice (**Figure 8A**). In Cfh−/− animals treated with a daily injection of human FH concentrate and sacrificed at 24 h, 48 h, 72 h, 96 h, and 120 h we found a progressive decrease of glomerular C3 staining, which was already significantly lower than in PBS-treated mice at 24 h (**Figures 8A, B**). Notably, the 120 h glomerular C3 staining in FH-treated Cfh−/− mice was not significantly different from staining in wild-type mice. These results indicate that the FH concentrate was effective in limiting C3 activation and also favored the clearance of C3 deposits from the glomerular tuft.

No tubular C3 staining was observed in PBS-treated Cfh−/− mice, while moderate peritubular C3 staining was observed in wild-type mice (p<0.05 vs. PBS-treated Cfh−/− mice, **Figure 8C**). The lack of tubular C3 staining in PBS-treated Cfh−/− mice likely reflected the massive C3 consumption in the circulation and the entrapment of C3 activation products within the glomerular structures. In Cfh−/− animals treated with a daily injection of human FH concentrate and sacrificed at 24 h, 48 h, 72 h, 96 h, and 120 h we found a progressive increase of tubular C3 staining, which was already significantly higher than in PBS-treated mice at 24h and paralleled the decrease of glomerular C3 staining. The appearance of C3 staining in the tubular compartment after FH administration in CFH -/- mice could be explained both by the mobilization of C3b inactivation products from the glomerulus due to the cofactor activity of injected FH and by the partial restoration of C3 levels in the circulation, so that C3 molecules were filtered by the glomeruli from the blood to tubular compartment.

Notably, the 72h and 120 h tubular C3 staining in FH-treated Cfh−/− mice was significantly higher than tubular C3 staining in wild-type mice (**Figures 8C, D**). Whether the higher-than-normal tubular staining in Cfh−/− mice treated with multiple injections of FH is due to tubular uptake of C3 activation products cleared from the glomeruli or reflects an immune response against the human FH concentrate remains to be established.

## Discussion

Over the past few years, plasma collection has been significantly reduced due to the COVID-19 pandemic. This has underscored the importance of optimizing plasma use for therapeutic development, a concern that has been at the forefront for both society and the industry. Annually, over 50 million liters of collected plasma are utilized for the production of immunoglobulin and albumin. This process incurs a total expenditure exceeding US$20 billion, highlighting the scale and economic impact of this sector (35).

Utilizing plasma intended for the production of higher-value products to develop new plasma-derived therapies may not be economically viable, especially for rare disease indications. Therefore, the use of discarded plasma fractionation intermediates, which are industrial waste generated during the manufacturing of medically valuable products, for the purification of other plasma proteins needed for various therapies, offers significant added value. Firstly, the ethical implications of using plasma, a scarce and precious resource derived from donations, for therapeutic development cannot be overlooked. Secondly, the cost-effectiveness of using plasma for the development of therapies for ultra-rare diseases could be questionable compared to the use of waste plasma, given the high cost of goods and the limited patient population. Thirdly, enhancing the economic feasibility of developing plasma-derived orphan drugs could positively impact the cost of rare disease treatments, benefiting patients and national health systems. Lastly, reducing the industrial waste generated in the plasma fractionation process by reintroducing such waste into the production cycle could lessen the industry’s environmental footprint. To address these aspects, we conducted a proteomic analysis of the primary unused fractionation intermediates from an industrial plasma fractionation plant and discovered FH in waste Fraction III (36).

We therefore developed a chromatography purification method for FH starting from this waste fraction. The purification steps were constantly monitored to obtain a product as pure and functional as possible. As FH can be cleaved by plasma proteinases during purification procedures, generating two fragments (130 and 35 kDa) we evaluated its integrity during all purification phases. Moreover, we used a cofactor assay to monitor the activity of purification intermediates, and the results were used to drive the procedure.

The purity of our final product was analyzed by proteomic analysis and by SDS-PAGE and Western blot. The presence of the splice variant FHL-1 was excluded using a specific monoclonal antibody.

The final product was further characterized with three different functional assays to ensure that the main properties of FH are preserved. Our final product is fully active as demonstrated by cofactor and decay acceleration assays and can attach to surface-bound C3b.FH circulates in plasma predominantly in the monomeric form, however, it was reported a weak tendency for dimer formation (37) which is greatly enhanced in the presence of polyanions (38). Several studies report that FH tends to oligomerize when stored at high concentrations in solution (39). In gel filtration experiments, FH migrates with an apparent size of 330,000 Da and was therefore often erroneously reported as a dimer. However, this characteristic is due to the high flexibility of the molecule which can even fold back on itself altering its mobility in agarose gel. Sedimentation equilibrium analysis by analytical ultracentrifugation demonstrated that this form corresponds to the monomer (38).

We analyzed our FH concentrate by Native PAGE and the results showed that it is predominantly in the monomeric form, with small percentages of higher molecular weight aggregates.

Before testing our product in a clinically relevant animal model, we investigated its *in vivo* biodistribution using PET/CT imaging. This was achieved by labeling the purified FH with ^18^F, utilizing a bifunctional conjugation strategy. This strategy is based on the azide-alkyne Cu-catalyzed 1,3-dipolar cycloaddition reaction, where the azide functional group was introduced on FH and the radionuclide was incorporated in short-chain, ^18^F-radiolabelled alkynes. Functionalization of FH was achieved by amide coupling, exploiting the lysine residues available (~80 residues in the sequence); while not chemiospecific, the average quantity and position of functionalization was sufficient to allow successful click chemistry conjugation with the radiofluorinated synthon.

Innovative reaction conditions were applied to the radiolabeling of the ^18^F-alkynes, which were optimized using a microfluidic system, that allowed to explore multiple consecutive reaction conditions, thus achieving the best RCY with minimal use of precious starting materials. Finally, the synthesis of the [^18^F]fluoropentyne intermediate did not require time-consuming chromatographic purifications and allowed a simple one-pot bioconjugation reaction which included the online precursor purification by distillation and the functionalized FH coupling reaction, affording the final compound [^18^F]FPe-(trN-PEG)@FH in good purity and yield.

Kinetics of FH distribution, as measured by imaging experiments, was shown to be different between normal and diseased mice. In particular, a point-wise comparison between the time activity curves (TACs) of the two groups revealed statistically significant differences in liver uptake in the time range of 20–55 min post-injection as well as in the kidneys in the time range of 30–40 min post injection. Even though the same statistical test did not report significant differences in the bladder and gallbladder TACs, the observed different shapes (in particular, their slope and interquartile ranges), the delayed bladder and gallbladder TACs of CFH- mice as compared to the control mice, along with the above-mentioned result about liver and kidney uptake, coherently suggest a dysfunction of the renal and hepatobiliary system in the Cfh-/- group. Also, a higher degree of intergroup variability for the [^18^F]FPe-(trN-PEG)@FH in almost all organs was found in diseased animals, as compared to control ones. The small number of animals used for the imaging experiments (n=5 for both groups) shall be regarded as a limitation of the present study. The characterization by PET/CT imaging and, to the best of the authors’ knowledge, the labelling and imaging of such an important molecule has never been reported with a PET tracer before, thus representing one of the main novelties of this study. Another limitation is the absence of a plasma stability test of the ^18^F-labeled FH tracer, which will be performed in future experiments.

Since FH is the main regulator of the alternative pathway of complement (40), we then assessed whether the FH concentrate was effective in correcting the severe complement alternative pathway dysregulation in Cfh-/- mice, a model of C3G (41). In these animals, C3 convertase activation in the fluid phase is unrestricted, leading to C3 consumption. Finding that the reduction in serum C3 levels observed in Cfh-/- mice was significantly limited by treatment with the FH concentrate, documented *in vivo* that the product possesses the expected complement regulatory activity. Intense glomerular deposits of C3 products are observed both in Cfh-/- mice and in patients with C3G (42), and this reflects hyperactivation of the complement alternative pathway. Indeed, when we analyzed kidney tissue through immunofluorescence experiments, we found marked staining of C3 in the capillary wall in Cfh-/- mice. Treatment with the FH concentrate was able to significantly reduce glomerular C3 staining in Cfh-/- mice which was notably almost completely normalized after five daily administrations of the FH concentrate. These results indicate that the FH concentrate was effective in limiting C3 activation and also favored the clearance of C3 deposits from the glomerular tuft.

## Conclusions

In summary, our findings provide important insights into the potential benefits of the use of discarded plasma fractionation intermediates for the purification of the natural complement inhibitor FH. PET/CT imaging was used to explore the *in vivo* biodistribution of FH, a protein that is part of a complex and highly regulated process and, to the best of authors’ knowledge, the labeling and imaging of such an important molecule has never been reported before, thus representing a relevant novelty of this study and a promising tool for future pharmacology assessment. All the results reported suggest that the obtained FH concentrate could represent a promising therapeutic approach for patients with C3G and FH deficiency (43), but also in more common C3G/MPGN patients with C3NeF-dependent C3 convertase stabilization, since FH accelerates the decay of the C3 convertase complex (44). Administration of exogenous FH could be useful also in other conditions associated with alternative pathway dysregulation ranging from the rare hematological disease paroxysmal nocturnal hemoglobinuria, to the common blinding disease age-related macular degeneration (3) Considering the human and economic cost of plasma-derived products and the shortage of blood and its derivatives, the use of plasma waste fractions for the therapy of orphan rare diseases will represent an ethical and essential approach.

## Data availability statement

The datasets presented in this study can be found in online repositories. The names of the repository/repositories and accession number(s) can be found below: PXD050268 (ProteomeXchange).

## Ethics statement

The studies involving animal participants were reviewed and approved by Dr. Silvia Burchielli, Centro Biomedicina Sperimentale, Area della Ricerca del CNR, Pisa. The study was conducted in accordance with the local legislation and institutional requirements.

## Author contributions

FM: Conceptualization, Data curation, Formal analysis, Investigation, Supervision, Writing – original draft, Writing – review & editing. GP: Methodology, Software, Writing – review & editing. SB: Investigation, Methodology, Writing – original draft, Writing – review & editing. AL: Investigation, Methodology, Writing – review & editing. DP: Methodology, Writing – review & editing. SR: Investigation, Methodology, Writing – review & editing. EC: Methodology, Writing – review & editing. FN: Writing – review & editing. AM: Writing – review & editing. RD: Writing – review & editing. AC: Investigation, Methodology, Writing – review & editing. CF: Data curation, Writing – review & editing, Conceptualization, Project administration, Resources. MN: Investigation, Methodology, Writing – review & editing, Formal analysis. PS: Data curation, Investigation, Methodology, Writing – review & editing. GR: Investigation, Methodology, Resources, Supervision, Validation, Writing – review & editing.
