# Supplementary material for: Proof of concept of a new plasma complement Factor H from waste plasma fraction
Source: Front Immunol. 2024 Jun 6;15:1334151. doi: 10.3389/fimmu.2024.1334151 (PMC11197005; doi:10.3389/fimmu.2024.1334151)
Supplement: Supplementary file 1 [file DataSheet_1.docx]

Supplementary Material

**Figure S1** : SDS-PAGE and Western Blot of purification intermediates under (A-B) non reducing and (C-D) reducing condition. Lane 1, molecular weight standard; Lane 2, filtration intermediate; Lane 3, Fractogel EMD DMAE eluate; Lane 4, AF-Heparin HC- 650M eluate; Lane 5, commercial FH (Merck)

In the Western blot under reducing condition (D) are visible three bands that identify, the integral form of FH, the larger fragment (130 kda) and smaller (35 kda) of the truncated form.


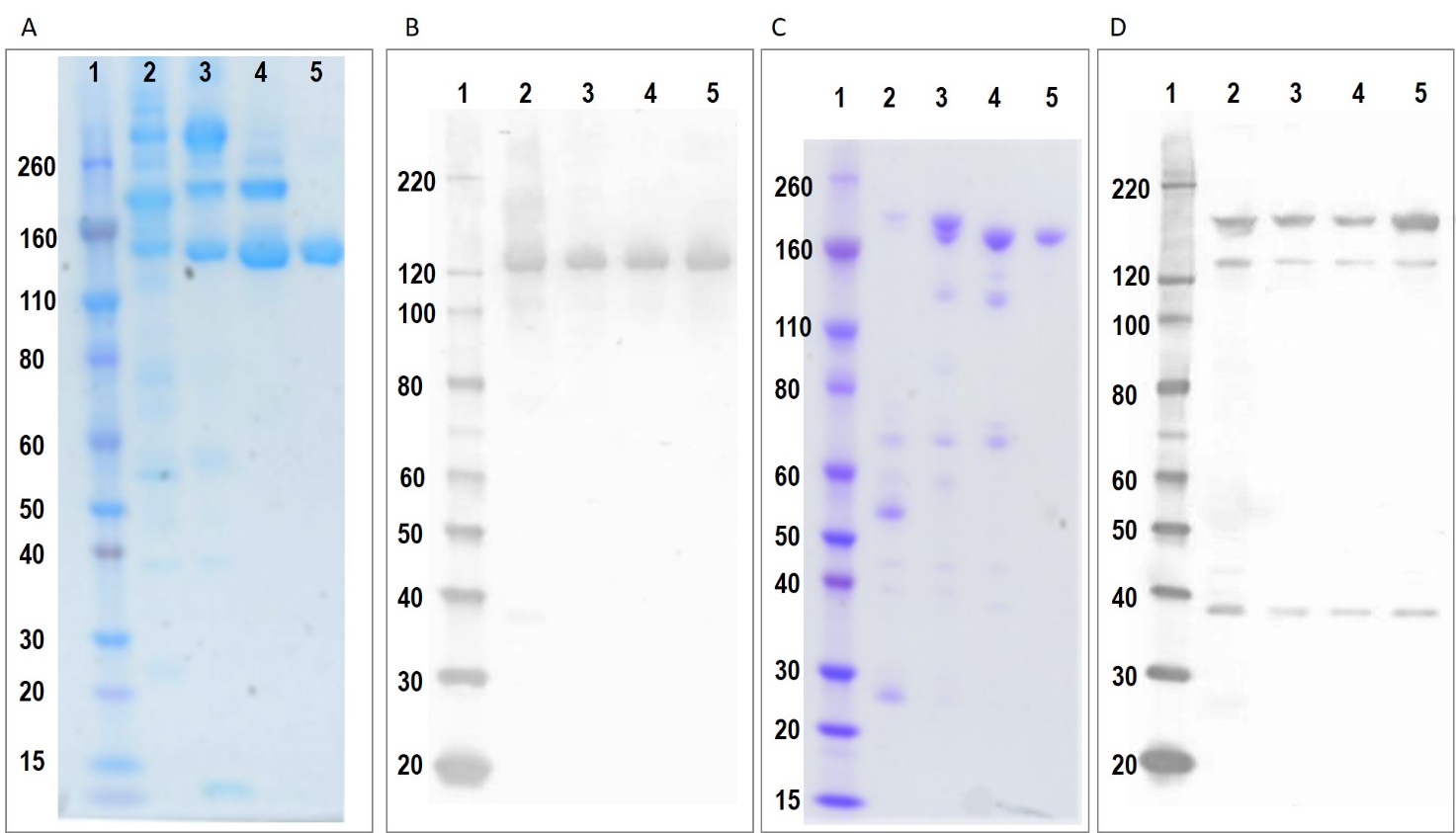


**Figure S2:** Representative graph of human FH levels in Cfh−/− mice until 120 h

|  | FH (μg/ml) | | | | |
| --- | --- | --- | --- | --- | --- |
| #mouse | 24 | 48 | 72 | 96 | 120 |
| T9 | 33.79 | 8.98 | 34.51 | 49.13 | 44.88 |
| T11A | 32.5 | 25.13 | 39.94 | 43.41 | 21.23 |
| T29 | 32.7 | 35.57 | 50.53 | 49.93 | died |
| T31 | 31.55 | 34.54 | 19.37 | 21.58 | died |
| mean | 32.64 | 26.06 | 36.09 | 41.01 | 33.06 |
| SD | 0.92 | 12.31 | 12.98 | 13.28 | 16.72 |

**Figure S3:** Reaction scheme for the labelling of FH using CuAAC click reaction


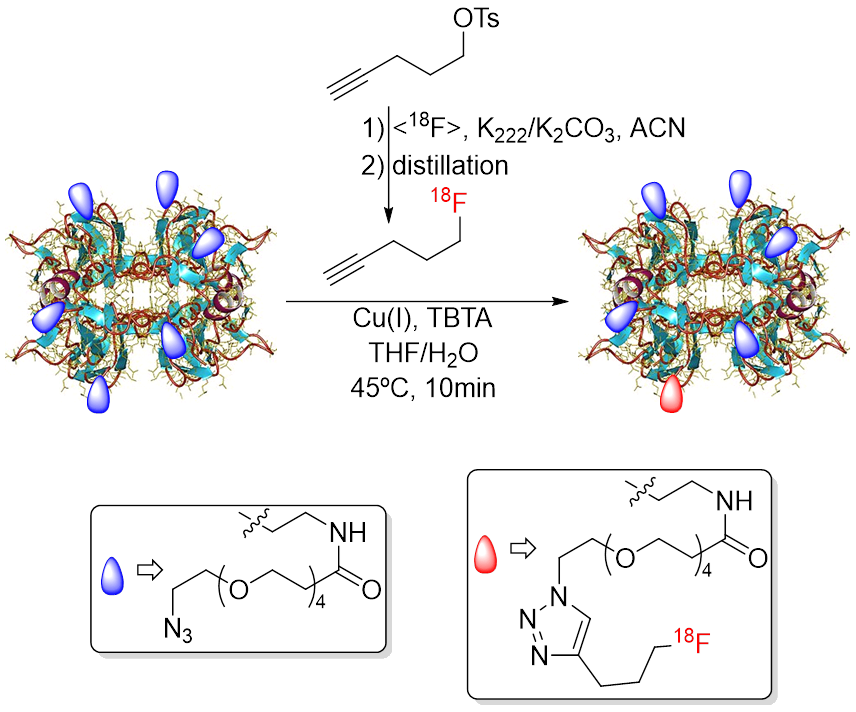


**Table S1:** Quantification of the truncated form of FH by SDS-PAGE and densitometry. The percentages were calculated from intensity of the bands corresponding to intact or cleaved form over total FH.

|  | **Without aprotinin** | **With aprotinin** |
| --- | --- | --- |
| Intact factor H | 90 % | 95 % |
| Cleaved factor H | 10 % | 5 % |

**Table S2**: Results of proteomic analysis of N=3 batches with their correspondence Factor H identifying peptides: **Batch N1 (code proteomeXchange: 16); Batch N2 (code proteomeXchange: 17); Batch N3 (code proteomeXchange: 18)**

**Batch N=1 (code:16)**

Tot: 211 proteins (95% confidence)

| **N** | **Accession** | **Description** | **Score** |
| --- | --- | --- | --- |
| 1 | P08603-1 | complement factor H | 1240.87 |
| 2 | P01024 | Complement C3 | 813.08 |
| 3 | P0C0L5 | Complement C4-B | 428.71 |
| 4 | P0C0L4-1 | Complement C4-A | 422.17 |
| 5 | P01031 | Complement C5 | 332.86 |
| 6 | P02751 | fibronectin | 326.92 |
| 7 | P01023 | alpha-2-macroglobulin | 268.61 |
| 8 | P01871 | immunoglobulin heavy constant mu | 267.71 |
| 9 | P04220 | Ig MU heavy chain disease protein | 164.92 |
| 10 | P04264 | Keratin, type II cytoskeletal 1 | 164.32 |
| 11 | P01834 | immunoglobulin kappa constant | 137.55 |
| 12 | P02768-1 | Serum albumin | 135.97 |
| 13 | P02675 | Fibrinogen beta chain | 131.65 |
| 14 | P13645 | Keratin, type I cytoskeletal 10 | 122.76 |
| 15 | P04114 | apolipoprotein B-100 | 121.53 |
| 16 | P0DOX7 | immunoglobulin kappa light chain | 121.32 |
| 17 | P35527 | Keratin, type I cytoskeletal 9 | 106.88 |
| 18 | P02679 | Fibrinogen gamma chain | 101.38 |
| 19 | P35908 | Keratin, type II cytoskeletal 2 epidermal | 81.16 |
| 20 | O43866 | CD5 antigen-like | 78.96 |
| 21 | P01876 | immunoglobulin heavy constant alpha 1 | 78.4 |
| 22 | P0DOY2 | immunoglobulin lambda constant 2 | 74.06 |
| 23 | P00760 | Cationic trypsin PE=1 SV=3 | 69.78 |
| 24 | P0DOX5 | immunoglobulin gamma-1 heavy chain | 62 |
| 25 | P02671-1 | Fibrinogen alpha chain | 61.87 |
| 26 | P0DOX8 | Immunoglobulin lambda-1 light chain | 60.29 |
| 27 | P04003 | C4b-binding protein alpha chain | 57.73 |
| 28 | P00736 | Complement C1r subcomponent | 54.45 |
| 29 | P00747 | Plasminogen | 52.98 |
| 30 | P00751-1 | Complement factor B | 50.91 |
| 31 | P02538 | Keratin, type II cytoskeletal 6A | 50.32 |
| 32 | P48668 | Keratin, type II cytoskeletal 6C | 49.98 |
| 33 | Q03591 | Complement factor H-related protein 1 | 49.96 |
| 34 | P13647 | keratin, type II cytoskeletal 5 | 47.17 |
| 35 | P08779 | Keratin, type I cytoskeletal 16 | 46.63 |
| 36 | Q5XQN5 | Keratin, type II cytoskeletal 5 GN=KRT5 PE=1 SV=1 | 44.36 |
| 37 | P02533 | Keratin, type I cytoskeletal 14 | 39.92 |
| 38 | Q922U2 | Keratin, type II cytoskeletal 5 GN=Krt5 PE=1 SV=1 | 39.25 |
| 39 | P01591 | Immunoglobulin J chain | 38.61 |
| 40 | P02769 | Serum albumin GN=ALB PE=1 SV=4 | 35.6 |
| 41 | P01860 | Immunoglobulin heavy constant gamma 3 | 31.15 |
| 42 | P00739-1 | Haptoglobin-related protein | 28.9 |
| 43 | P09871 | Complement C1s subcomponent | 28.62 |
| 44 | P07357 | Complement component C8 alpha chain | 27.88 |
| 45 | Q02985-1 | Complement factor H-related protein 3 | 27.43 |
| 46 | P19827-1 | Inter-alpha-trypsin inhibitor heavy chain H1 | 27.13 |
| 47 | P10909-1 | Clusterin | 26.88 |
| 48 | Q04695 | Keratin, type I cytoskeletal 17 | 24.73 |
| 49 | P19823 | Inter-alpha-trypsin inhibitor heavy chain H2 | 24.29 |
| 50 | P04004 | Vitronectin | 23.85 |
| 51 | Q14624-1 | Inter-alpha-trypsin inhibitor heavy chain H4 | 23.46 |
| 52 | P00738 | Haptoglobin | 22.66 |
| 53 | P01861 | Immunoglobulin heavy constant gamma 4 | 21.76 |
| 54 | P01833 | Polymeric immunoglobulin receptor | 21.07 |
| 55 | P35858 | Insulin-like growth factor-binding protein complex acid labile subunit | 18.38 |
| 56 | P0DOX2 | Immunoglobulin alpha-2 heavy chain | 18.11 |
| 57 | P02748 | complement component C9 | 15.32 |
| 58 | P05546 | Heparin cofactor 2 | 14.86 |
| 59 | A0A0C4DH72 | immunoglobulin kappa variable 1-6 | 14.11 |
| 60 | P01859 | Immunoglobulin heavy constant gamma 2 | 13.89 |
| 61 | P01619 | Immunoglobulin kappa variable 3-20 | 12.91 |
| 62 | P29622 | Kallistatin | 12.75 |
| 63 | P22105 | tenascin-X | 12.35 |
| 64 | A0A0B4J1X5 | immunoglobulin heavy variable 3-74 | 12.32 |
| 65 | P27918 | Properdin | 12.17 |
| 66 | P11226 | Mannose-binding protein C | 11.85 |
| 67 | P04196 | Histidine-rich glycoprotein | 11.08 |
| 68 | P03952 | Plasma kallikrein | 10.69 |
| 69 | P01009-1 | alpha-1-antitrypsin | 10.17 |
| 70 | P49908 | Selenoprotein P | 9.85 |
| 71 | P81605 | Dermcidin | 9.39 |
| 72 | P07360 | Complement component C8 gamma chain | 9.36 |
| 73 | P07358 | Complement component C8 beta chain | 9.21 |
| 74 | P19652 | Alpha-1-acid glycoprotein 2 | 9.08 |
| 75 | P27169 | Serum paraoxonase/arylesterase 1 | 9.02 |
| 76 | P01780 | Immunoglobulin heavy variable 3-7 | 8.91 |
| 77 | P06331 | immunoglobulin heavy variable 4-34 | 8.85 |
| 78 | P68871 | Hemoglobin subunit beta | 8.63 |
| 79 | P02790 | Hemopexin | 8.54 |
| 80 | P02760 | Protein AMBP | 8.49 |
| 81 | P01701 | immunoglobulin lambda variable 1-51 | 8.08 |
| 82 | A0A075B6S5 | immunoglobulin kappa variable 1-27 | 7.9 |
| 83 | Q13885 | Tubulin beta-2A chain | 7.8 |
| 84 | P50607 | Tubby protein homolog | 7.8 |
| 85 | P01599 | Immunoglobulin kappa variable 1-17 | 7.66 |
| 86 | P02747 | Complement C1q subcomponent subunit C | 7.58 |
| 87 | A0A0A0MRZ8 | immunoglobulin kappa variable 3D-11 | 7.52 |
| 88 | P80748 | Immunoglobulin lambda variable 3-21 | 7.34 |
| 89 | P00748 | Coagulation factor XII | 7.22 |
| 90 | O75636-1 | Ficolin-3 | 7.19 |
| 91 | A0A087WSY6 | Immunoglobulin kappa variable 3D-15 | 7.18 |
| 92 | P01042 | kininogen-1 | 7.07 |
| 93 | P01602 | Immunoglobulin kappa variable 1-5 | 6.87 |
| 94 | P13671 | Complement component c6 | 6.82 |
| 95 | A0A0C4DH38 | immunoglobulin heavy variable 5-51 | 6.62 |
| 96 | P06312 | immunoglobulin kappa variable 4-1 | 6.22 |
| 97 | P01700 | Immunoglobulin lambda variable 1-47 | 6.21 |
| 98 | Q9P2C4 | Transmembrane protein 181 | 6.21 |
| 99 | P01768 | Immunoglobulin heavy variable 3-30 | 6.05 |
| 100 | P0DP04 | Immunoglobulin heavy variable 3-43D | 6.04 |
| 101 | A0A0J9YX35 | immunoglobulin heavy variable 3-64D | 5.9 |
| 102 | Q9UK55 | Protein Z-dependent protease inhibitor | 5.85 |
| 103 | A0A0A0MS15 | immunoglobulin heavy variable 3-49 | 5.8 |
| 104 | Q16777 | Histone H2A type 2-C | 5.8 |
| 105 | Q02413 | Desmoglein-1 | 5.7 |
| 106 | Q92954-1 | Proteoglycan 4 | 5.57 |
| 107 | P25311 | Zinc-alpha-2-glycoprotein | 5.56 |
| 108 | P01011-1 | Alpha-1-antichymotrypsin | 5.47 |
| 109 | P06396 | Gelsolin | 5.41 |
| 110 | P01743 | immunoglobulin heavy variable 1-46 | 5.4 |
| 111 | P15924-1 | Desmoplakin | 5.24 |
| 112 | O75475-1 | PC4 and SFRS1-interacting protein | 5.16 |
| 113 | P20851 | C4b-binding protein beta chain | 5.04 |
| 114 | P01709 | Immunoglobulin lambda variable 2-8 | 4.91 |
| 115 | P01601 | Immunoglobulin kappa variable 1D-16 | 4.57 |
| 116 | Q2UY09 | Collagen alpha-1(XXVIII) chain | 4.5 |
| 117 | P07225 | Vitamin K-dependent protein S | 4.46 |
| 118 | P02649 | Apolipoprotein E | 4.3 |
| 119 | P01019 | Angiotensinogen | 4.23 |
| 120 | P04430 | Immunoglobulin kappa variable 1-16 | 4.18 |
| 121 | Q86X10-1 | Ral GTPase-activating protein subunit beta | 4.15 |
| 122 | A0A0B4J1U7 | immunoglobulin heavy variable 6-1 | 4.13 |
| 123 | P01717 | Immunoglobulin lambda variable 3-25 | 4.11 |
| 124 | P62987 | Ubiquitin-60S ribosomal protein L40 | 4.09 |
| 125 | P02647 | Apolipoprotein A-I | 4.09 |
| 126 | P35611-1 | Alpha-adducin | 4.07 |
| 127 | A0A075B6K4 | immunoglobulin lambda variable 3-10 | 4.05 |
| 128 | A0A0B4J2D9 | immunoglobulin kappa variable 1D-13 | 3.78 |
| 129 | O00187-1 | Mannan-binding lectin serine protease 2 | 3.75 |
| 130 | A0A0C4DH68 | immunoglobulin kappa variable 2-24 | 3.71 |
| 131 | P69905 | Hemoglobin subunit alpha | 3.68 |
| 132 | Q8N8A6 | ATP-dependent RNA helicase DDX51 | 3.63 |
| 133 | Q8IWX7 | Protein unc-45 homolog B | 3.43 |
| 134 | P06702 | Protein S100-A9 | 3.42 |
| 135 | P05155 | Plasma protease C1 inhibitor | 3.37 |
| 136 | Q96HY6-1 | DDRGK domain-containing protein 1 | 3.36 |
| 137 | A0A075B6I4 | immunoglobulin lambda variable 10-54 | 3.35 |
| 138 | P23083 | Immunoglobulin heavy variable 1-2 | 3.32 |
| 139 | P01594 | Immunoglobulin kappa variable 1-33 | 3.3 |
| 140 | A2RRH5-1 | WD repeat-containing protein 27 | 3.25 |
| 141 | Q9HCG8 | Pre-mRNA-splicing factor CWC22 homolog | 3.23 |
| 142 | A0A0B4J1V0 | Immunoglobulin heavy variable 3-15 | 3.2 |
| 143 | Q6ZUJ4 | Uncharacterized protein C3orf62 | 3.12 |
| 144 | Q00169 | Phosphatidylinositol transfer protein alpha isoform | 3.1 |
| 145 | P10599-1 | thioredoxin | 3.1 |
| 146 | P00973-1 | 2'-5'-oligoadenylate synthase 1 | 3.08 |
| 147 | P02766 | Transthyretin | 3.07 |
| 148 | Q9NY65 | Tubulin alpha-8 chain | 3.05 |
| 149 | B1ANY3 | Putative protein FAM220BP | 3.03 |
| 150 | Q96RP9 | Elongation factor G, mitochondrial | 3.01 |
| 151 | Q5VV43 | Dyslexia-associated protein KIAA0319 | 3.01 |
| 152 | P01880 | Immunoglobulin heavy constant delta | 2.99 |
| 153 | P06310 | Immunoglobulin kappa variable 2-30 | 2.98 |
| 154 | O75909-1 | Isoform 3 of Cyclin-K | 2.96 |
| 155 | Q9H7S9 | Zinc finger protein 703 | 2.96 |
| 156 | P52848-1 | Bifunctional heparan sulfate N-deacetylase/N-sulfotransferase 1 | 2.95 |
| 157 | Q01167 | Forkhead box protein K2 | 2.92 |
| 158 | A5A3E0 | POTE ankyrin domain family member F | 2.91 |
| 159 | Q9UPX8 | SH3 and multiple ankyrin repeat domains protein 2 | 2.89 |
| 160 | Q9BV20-1 | methylthioribose-1-phosphate isomerase | 2.87 |
| 161 | A0A0A0MT36 | Immunoglobulin kappa variable 6D-21 | 2.85 |
| 162 | Q58HT5 | Acyl-CoA wax alcohol acyltransferase 1 | 2.85 |
| 163 | Q8TB05-2 | Isoform 2 of UBA-like domain-containing protein 1 | 2.84 |
| 164 | Q8WYN0 | Cysteine protease ATG4A | 2.84 |
| 165 | Q53F19 | Nuclear cap-binding protein subunit 3 | 2.84 |
| 166 | A0A075B6I1 | immunoglobulin lambda variable 4-60 | 2.83 |
| 167 | Q8NC56-1 | LEM domain-containing protein 2 | 2.82 |
| 168 | A0A0G2JS06 | Immunoglobulin lambda variable 5-39 | 2.79 |
| 169 | Q9H7U1 | serine-rich coiled-coil domain-containing protein 2 | 2.79 |
| 170 | Q6P6C2-1 | Isoform 1 of RNA demethylase ALKBH5 | 2.74 |
| 171 | Q96JG9 | Zinc finger protein 469 | 2.74 |
| 172 | Q9Y2H9 | Microtubule-associated serine/threonine-protein kinase 1 | 2.7 |
| 173 | Q8NEU8-1 | DCC-interacting protein 13-beta | 2.69 |
| 174 | P05109 | Protein S100-A8 | 2.62 |
| 175 | Q96MG2 | Junctional sarcoplasmic reticulum protein 1 | 2.62 |
| 176 | O15230 | Laminin subunit alpha-5 | 2.62 |
| 177 | Q86UB9 | transmembrane protein 135 | 2.61 |
| 178 | P56199 | Integrin alpha-1 | 2.61 |
| 179 | Q6PCT2-1 | F-box/LRR-repeat protein 19 | 2.6 |
| 180 | Q8NCQ7 | Protein PROCA1 | 2.57 |
| 181 | Q8WUP2 | Filamin-binding LIM protein 1 | 2.51 |
| 182 | Q9UPZ3 | Hermansky-Pudlak syndrome 5 protein | 2.46 |
| 183 | Q8N7P7 | Uncharacterized protein FLJ40521 | 2.45 |
| 184 | Q9HCH3 | Copine-5 | 2.43 |
| 185 | Q8WWM7-1 | ataxin-2-like protein | 2.43 |
| 186 | O60427 | Fatty acid desaturase 1 | 2.41 |
| 187 | Q28085 | Complement factor H GN=CFH PE=1 SV=3 | 2.41 |
| 188 | Q96PD5 | N-acetylmuramoyl-L-alanine amidase | 2.36 |
| 189 | A1L188 | NADH dehydrogenase [ubiquinone] 1 alpha subcomplex assembly factor 8 | 2.34 |
| 190 | Q92847-1 | Growth hormone secretagogue receptor type 1 | 2.34 |
| 191 | Q5JQC4 | cancer/testis antigen 47A | 2.3 |
| 192 | P02745 | Complement C1q subcomponent subunit A | 2.3 |
| 193 | P08670 | Vimentin | 2.3 |
| 194 | P01714 | Immunoglobulin lambda variable 3-19 | 2.27 |
| 195 | P02765 | Alpha-2-HS-glycoprotein | 2.27 |
| 196 | Q9Y283-1 | Inversin | 2.27 |
| 197 | O14791 | Apolipoprotein L1 | 2.21 |
| 198 | A0A075B6J9 | immunoglobulin lambda variable 2-18 | 2.19 |
| 199 | Q14247-1 | Src substrate cortactin | 2.19 |
| 200 | P07477 | Trypsin-1 | 2.17 |
| 201 | P62805 | histone H4 | 2.13 |
| 202 | Q15582 | Transforming growth factor-beta-induced protein ig-h3 | 2.13 |
| 203 | Q9BTV5 | fibronectin type III and SPRY domain-containing protein 1 | 2.08 |
| 204 | A0A075B6I9 | Immunoglobulin lambda variable 7-46 | 2.07 |
| 205 | Q9ULD2-6 | Isoform 6 of Microtubule-associated tumor suppressor 1 | 2.05 |
| 206 | P22532 | small proline-rich protein 2D | 2.03 |
| 207 | Q6ZWH5-1 | Serine/threonine-protein kinase Nek10 | 2.03 |
| 208 | Q8ND30 | Liprin-beta-2 | 2.02 |
| 209 | Q9BXU0 | Testis-expressed protein 12 | 1.98 |
| 210 | Q06033-1 | Inter-alpha-trypsin inhibitor heavy chain H3 | 1.98 |
| 211 | Q9NRH1-2 | Isoform 2 of Yae1 domain-containing protein 1 | 1.93 |

OS=Homo Sapiens

**Peptide List Batch n=1**

| **Accession** | **Description** | **Coverage [%]** | **# Peptides** | **Score Sequest HT** |
| --- | --- | --- | --- | --- |
| *P08603-1* | *complement factor H* | *73* | *82* | *1240.87* |
|  | Sequence |  |  | Score Sequest HT |
|  | AGEQVTYTCATYYK |  |  | 4.3 |
|  | AQTTVTCMENGWSPTPR |  |  | 3.76 |
|  | AQTTVTCMENGWSPTPR |  |  | 3.59 |
|  | AVYTCNEGYQLLGEINYR |  |  | 5.12 |
|  | CFEGFGIDGPAIAK |  |  | 2.95 |
|  | CFEGFGIDGPAIAKCLGEK |  |  | 4.02 |
|  | CGPPPPIDNGDITSFPLSVYAPASSVEYQCQNLYQLEGNK |  |  | 8.6 |
|  | CGPPPPIDNGDITSFPLSVYAPASSVEYQCQNLYQLEGNKR |  |  | 8.35 |
|  | CLHPCVISR |  |  | 2.31 |
|  | CLPVTAPENGK |  |  | 2.37 |
|  | CNMGYEYSER |  |  | 3.28 |
|  | CNMGYEYSER |  |  | 2.61 |
|  | CTLKPCDYPDIK |  |  | 3.48 |
|  | CTSTGWIPAPR |  |  | 3.54 |
|  | CVEISCKSPDVINGSPISQK |  |  | 4.46 |
|  | CYFPYLENGYNQNYGR |  |  | 4.45 |
|  | DGEKVSVLCQENYLIQEGEEITCK |  |  | 3.92 |
|  | DGEKVSVLCQENYLIQEGEEITCKDGR |  |  | 6.29 |
|  | DGWSAQPTCIK |  |  | 2.58 |
|  | DTSCVNPPTVQNAYIVSR |  |  | 5.57 |
|  | ECDTDGWTNDIPICEVVK |  |  | 3.08 |
|  | ECELPKIDVHLVPDR |  |  | 3.23 |
|  | EEYGHSEVVEYYCNPR |  |  | 5.12 |
|  | EFDHNSNIR |  |  | 2.45 |
|  | EGWIHTVCINGR |  |  | 2.83 |
|  | EKTKEEYGHSEVVEYYCNPR |  |  | 5.86 |
|  | EIMENYNIALR |  |  | 2.74 |
|  | EIMENYNIALR |  |  | 2.53 |
|  | EQVQSCGPPPELLNGNVK |  |  | 4.7 |
|  | FSCKPGFTIVGPNSVQCYHFGLSPDLPICK |  |  | 4.73 |
|  | FVCNSGYK |  |  | 2.07 |
|  | FVCNSGYKIEGDEEMHCSDDGFWSK |  |  | 4.43 |
|  | FVCNSGYKIEGDEEMHCSDDGFWSK |  |  | 3.35 |
|  | FVQGKSIDVACHPGYALPK |  |  | 3.1 |
|  | GDAVCTESGWRPLPSCEEK |  |  | 4.69 |
|  | GEWVALNPLR |  |  | 2.39 |
|  | GEWVALNPLRK |  |  | 2.83 |
|  | GKEGWIHTVCINGR |  |  | 4.44 |
|  | HGGLYHENMR |  |  | 3.67 |
|  | HGGLYHENMR |  |  | 2.71 |
|  | HRTGDEITYQCR |  |  | 3.9 |
|  | KCYFPYLENGYNQNYGR |  |  | 5.05 |
|  | KEFDHNSNIR |  |  | 3.72 |
|  | KGEWVALNPLR |  |  | 3.7 |
|  | KGEWVALNPLRK |  |  | 3.34 |
|  | IDVHLVPDR |  |  | 2.38 |
|  | IDVHLVPDRK |  |  | 2.93 |
|  | IEGDEEMHCSDDGFWSK |  |  | 4.8 |
|  | IEGDEEMHCSDDGFWSK |  |  | 3.96 |
|  | LGYVTADGETSGSITCGK |  |  | 4.32 |
|  | LGYVTADGETSGSITCGKDGWSAQPTCIK |  |  | 4.83 |
|  | IIYKENER |  |  | 2.15 |
|  | IIYKENERFQYK |  |  | 2.95 |
|  | IPCSQPPQIEHGTINSSR |  |  | 3.34 |
|  | LSYTCEGGFR |  |  | 2.28 |
|  | IVSSAMEPDR |  |  | 2.31 |
|  | IVSSAMEPDR |  |  | 2.23 |
|  | IVSSAMEPDREYHFGQAVR |  |  | 5.29 |
|  | IVSSAMEPDREYHFGQAVR |  |  | 5.1 |
|  | NDFTWFK |  |  | 2.37 |
|  | NGFYPATR |  |  | 2.42 |
|  | NGQWSEPPK |  |  | 1.92 |
|  | NTEILTGSWSDQTYPEGTQAIYK |  |  | 4.76 |
|  | RNTEILTGSWSDQTYPEGTQAIYK |  |  | 6.31 |
|  | RPCGHPGDTPFGTFTLTGGNVFEYGVK |  |  | 7.33 |
|  | RPYFPVAVGK |  |  | 3.1 |
|  | SCDIPVFMNAR |  |  | 3.19 |
|  | SCDIPVFMNAR |  |  | 1.91 |
|  | SCDNPYIPNGDYSPLR |  |  | 4.62 |
|  | SLGNVIMVCR |  |  | 2.96 |
|  | SLGNVIMVCR |  |  | 2.17 |
|  | SITCIHGVWTQLPQCVAIDK |  |  | 5.56 |
|  | SITCIHGVWTQLPQCVAIDKLK |  |  | 5.53 |
|  | SPDVINGSPISQK |  |  | 3.64 |
|  | SPPEISHGVVAHMSDSYQYGEEVTYK |  |  | 8.55 |
|  | SPPEISHGVVAHMSDSYQYGEEVTYK |  |  | 3.61 |
|  | SSIDIENGFISESQYTYALK |  |  | 4.96 |
|  | SSNLIILEEHLK |  |  | 3.87 |
|  | SSNLIILEEHLKNKK |  |  | 4.09 |
|  | SSQESYAHGTK |  |  | 2.14 |
|  | TDCLSLPSFENAIPMGEK |  |  | 3.34 |
|  | TDCLSLPSFENAIPMGEK |  |  | 2.95 |
|  | TGDEITYQCR |  |  | 2.93 |
|  | TGESVEFVCK |  |  | 3.02 |
|  | TGESVEFVCKR |  |  | 4.17 |
|  | TKEEYGHSEVVEYYCNPR |  |  | 6.46 |
|  | TKNDFTWFK |  |  | 3.1 |
|  | TTCWDGKLEYPTCAK |  |  | 3.8 |
|  | VSVLCQENYLIQEGEEITCK |  |  | 4.41 |
|  | VSVLCQENYLIQEGEEITCKDGR |  |  | 5.07 |
|  | WQSIPLCVEK |  |  | 2.23 |
|  | WSHPPSCIK |  |  | 2.51 |
|  | WSSPPQCEGLPCK |  |  | 3.21 |
|  | YYSYYCDEHFETPSGSYWDHIHCTQDGWSPAVPCLR |  |  | 4.37 |

**Batch N=2 (code 17)**

Tot: 157 proteins (95% confidence)

| N | Accession | Description | Score |
| --- | --- | --- | --- |
| 1 | P08603-1 | complement factor H | 1135.38 |
| 2 | P01024 | Complement C3 | 851.56 |
| 3 | P01031 | Complement C5 | 386.72 |
| 4 | P0C0L4-1 | Complement C4-A | 270.31 |
| 5 | P0C0L5 | Complement C4-B | 265.38 |
| 6 | P02751 | fibronectin | 257.38 |
| 7 | P01023 | alpha-2-macroglobulin | 251.16 |
| 8 | P01871 | immunoglobulin heavy constant mu | 234.39 |
| 9 | P04114 | apolipoprotein B-100 | 158.61 |
| 10 | P04220 | Ig MU heavy chain disease protein | 155.31 |
| 11 | P01834 | immunoglobulin kappa constant | 130.69 |
| 12 | P0DOX7 | immunoglobulin kappa light chain | 121.9 |
| 13 | P02675 | Fibrinogen beta chain | 106.57 |
| 14 | P02679 | Fibrinogen gamma chain | 106.2 |
| 15 | P02768-1 | Serum albumin | 76.44 |
| 16 | P01876 | immunoglobulin heavy constant alpha 1 | 75.22 |
| 17 | O43866 | CD5 antigen-like | 66.48 |
| 18 | P02671-1 | Fibrinogen alpha chain | 65.96 |
| 19 | Q03591 | Complement factor H-related protein 1 | 64.08 |
| 20 | P00747 | Plasminogen | 62.92 |
| 21 | P00760 | Cationic trypsin PE=1 SV=3 | 58.67 |
| 22 | P04264 | Keratin, type II cytoskeletal 1 | 58.5 |
| 23 | P01857 | Immunoglobulin heavy constant gamma 1 | 57.07 |
| 24 | P0DOY2 | immunoglobulin lambda constant 2 | 55.25 |
| 25 | P0DOX8 | Immunoglobulin lambda-1 light chain | 50.35 |
| 26 | P00736 | Complement C1r subcomponent | 49.8 |
| 27 | P35527 | Keratin, type I cytoskeletal 9 | 46.06 |
| 28 | P04196 | Histidine-rich glycoprotein | 44.35 |
| 29 | P13645 | Keratin, type I cytoskeletal 10 | 43.54 |
| 30 | P35858 | Insulin-like growth factor-binding protein complex acid labile subunit | 41.47 |
| 31 | P19827-1 | Inter-alpha-trypsin inhibitor heavy chain H1 | 34.33 |
| 32 | P01860 | Immunoglobulin heavy constant gamma 3 | 33.03 |
| 33 | P01591 | Immunoglobulin J chain | 32.13 |
| 34 | P19823 | Inter-alpha-trypsin inhibitor heavy chain H2 | 31.79 |
| 35 | Q14624-1 | Inter-alpha-trypsin inhibitor heavy chain H4 | 28.76 |
| 36 | P10909-1 | Clusterin | 28.6 |
| 37 | P00738 | Haptoglobin | 27.47 |
| 38 | P07357 | Complement component C8 alpha chain | 26.01 |
| 39 | Q02985-1 | Complement factor H-related protein 3 | 24.85 |
| 40 | P29622 | Kallistatin | 24.76 |
| 41 | P00739-1 | Haptoglobin-related protein | 23.95 |
| 42 | P35908 | Keratin, type II cytoskeletal 2 epidermal | 22.28 |
| 43 | P04003 | C4b-binding protein alpha chain | 22.08 |
| 44 | P19652 | Alpha-1-acid glycoprotein 2 | 19.08 |
| 45 | P02748 | complement component C9 | 18.87 |
| 46 | P01833 | Polymeric immunoglobulin receptor | 18.36 |
| 47 | P0DOX2 | Immunoglobulin alpha-2 heavy chain | 17.7 |
| 48 | P68871 | Hemoglobin subunit beta | 17.69 |
| 49 | P01030 | Complement C4 (Fragments) GN=C4 PE=1 SV=2 | 15.69 |
| 50 | P03952 | Plasma kallikrein | 15.42 |
| 51 | P01859 | Immunoglobulin heavy constant gamma 2 | 13.49 |
| 52 | P09871 | Complement C1s subcomponent | 13.04 |
| 53 | P00751-1 | Complement factor B | 12.97 |
| 54 | P27918 | Properdin | 12.32 |
| 55 | A0A0B4J1X5 | immunoglobulin heavy variable 3-74 | 12.24 |
| 56 | P00748 | Coagulation factor XII | 12.16 |
| 57 | P02760 | Protein AMBP | 12.14 |
| 58 | P02769 | Serum albumin GN=ALB PE=1 SV=4 | 11.97 |
| 59 | P01619 | Immunoglobulin kappa variable 3-20 | 11.15 |
| 60 | P69905 | Hemoglobin subunit alpha | 10.7 |
| 61 | P22105 | tenascin-X | 10.34 |
| 62 | Q92954-1 | Proteoglycan 4 | 10.19 |
| 63 | P49908 | Selenoprotein P | 10.1 |
| 64 | P02790 | Hemopexin | 10.04 |
| 65 | P13671 | Complement component c6 | 9.73 |
| 66 | P07358 | Complement component C8 beta chain | 9.5 |
| 67 | P06396 | Gelsolin | 9.42 |
| 68 | P01009-1 | alpha-1-antitrypsin | 9.33 |
| 69 | A0A0C4DH72 | immunoglobulin kappa variable 1-6 | 9.18 |
| 70 | P01780 | Immunoglobulin heavy variable 3-7 | 8.91 |
| 71 | P06312 | immunoglobulin kappa variable 4-1 | 8.8 |
| 72 | P04259 | keratin, type II cytoskeletal 6B | 8.28 |
| 73 | P04004 | Vitronectin | 8.24 |
| 74 | P07360 | Complement component C8 gamma chain | 8.19 |
| 75 | P01011-1 | Alpha-1-antichymotrypsin | 7.86 |
| 76 | P01019 | Angiotensinogen | 7.74 |
| 77 | A0A075B6S5 | immunoglobulin kappa variable 1-27 | 7.5 |
| 78 | A0A0B4J1U7 | immunoglobulin heavy variable 6-1 | 7.49 |
| 79 | P27169 | Serum paraoxonase/arylesterase 1 | 7.34 |
| 80 | P80748 | Immunoglobulin lambda variable 3-21 | 7.03 |
| 81 | Q06033-1 | Inter-alpha-trypsin inhibitor heavy chain H3 | 6.99 |
| 82 | P04430 | Immunoglobulin kappa variable 1-16 | 6.98 |
| 83 | P01599 | Immunoglobulin kappa variable 1-17 | 6.82 |
| 84 | P06331 | immunoglobulin heavy variable 4-34 | 6.71 |
| 85 | P01700 | Immunoglobulin lambda variable 1-47 | 6.52 |
| 86 | P01042 | kininogen-1 | 6.43 |
| 87 | P0DP04 | Immunoglobulin heavy variable 3-43D | 6.06 |
| 88 | A0A0A0MS15 | immunoglobulin heavy variable 3-49 | 6.02 |
| 89 | A0A0C4DH38 | immunoglobulin heavy variable 5-51 | 5.95 |
| 90 | P05546 | Heparin cofactor 2 | 5.6 |
| 91 | P02649 | Apolipoprotein E | 5.49 |
| 92 | Q5XQN5 | Keratin, type II cytoskeletal 5 GN=KRT5 PE=1 SV=1 | 5.46 |
| 93 | P50607 | Tubby protein homolog | 5.36 |
| 94 | P02745 | Complement C1q subcomponent subunit A | 5.07 |
| 95 | P02765 | Alpha-2-HS-glycoprotein | 4.92 |
| 96 | Q5SNV9 | Uncharacterized protein C1orf167 | 4.83 |
| 97 | P07225 | Vitamin K-dependent protein S | 4.8 |
| 98 | Q53F19 | Nuclear cap-binding protein subunit 3 | 4.27 |
| 99 | P01624 | Immunoglobulin kappa variable 3-15 | 4.03 |
| 100 | A0A0B4J2D9 | immunoglobulin kappa variable 1D-13 | 4.02 |
| 101 | Q9UK55 | Protein Z-dependent protease inhibitor | 3.91 |
| 102 | Q9H4Q3 | PR domain zinc finger protein 13 | 3.71 |
| 103 | P05155 | Plasma protease C1 inhibitor | 3.63 |
| 104 | Q86TB3 | Alpha-protein kinase 2 | 3.6 |
| 105 | Q13885 | Tubulin beta-2A chain | 3.58 |
| 106 | Q8TDW4-1 | Suppressor of tumorigenicity 7 protein-like | 3.56 |
| 107 | A0A0C4DH68 | immunoglobulin kappa variable 2-24 | 3.44 |
| 108 | Q8N8A6 | ATP-dependent RNA helicase DDX51 | 3.37 |
| 109 | Q9BUY5 | zinc finger protein 426 | 3.33 |
| 110 | P01742 | Immunoglobulin heavy variable 1-69 | 3.31 |
| 111 | P01594 | Immunoglobulin kappa variable 1-33 | 3.26 |
| 112 | P02747 | Complement C1q subcomponent subunit C | 3.23 |
| 113 | P10643 | Complement component C7 | 3.12 |
| 114 | O95071 | E3 ubiquitin-protein ligase UBR5 | 2.99 |
| 115 | P08697-1 | Alpha-2-antiplasmin | 2.95 |
| 116 | Q96RN5 | Mediator of RNA polymerase II transcription subunit 15 | 2.94 |
| 117 | Q9P2C4 | Transmembrane protein 181 | 2.94 |
| 118 | P00973-1 | 2'-5'-oligoadenylate synthase 1 | 2.94 |
| 119 | Q04756 | Hepatocyte growtH factor activator | 2.9 |
| 120 | P04626-6 | Isoform 6 of Receptor tyrosine-protein kinase erbB-2 | 2.86 |
| 121 | P57105 | Synaptojanin-2-binding protein | 2.84 |
| 122 | Q9H4M7-1 | Pleckstrin homology domain-containing family A member 4 | 2.8 |
| 123 | O75636-1 | Ficolin-3 | 2.75 |
| 124 | A0A0A0MT36 | Immunoglobulin kappa variable 6D-21 | 2.75 |
| 125 | A0A075B6I1 | immunoglobulin lambda variable 4-60 | 2.67 |
| 126 | Q2TAC6 | Kinesin-like protein KIF19 | 2.63 |
| 127 | P01701 | immunoglobulin lambda variable 1-51 | 2.62 |
| 128 | Q9UMD9-1 | Collagen alpha-1(XVII) chain | 2.6 |
| 129 | Q8TDW7 | Protocadherin Fat 3 | 2.58 |
| 130 | A0A0B4J1V0 | Immunoglobulin heavy variable 3-15 | 2.57 |
| 131 | A0A0A0MRZ8 | immunoglobulin kappa variable 3D-11 | 2.55 |
| 132 | P01714 | Immunoglobulin lambda variable 3-19 | 2.53 |
| 133 | Q15582 | Transforming growth factor-beta-induced protein ig-h3 | 2.45 |
| 134 | A2NJV5 | Immunoglobulin kappa variable 2-29 | 2.42 |
| 135 | P81605 | Dermcidin | 2.41 |
| 136 | P07477 | Trypsin-1 | 2.4 |
| 137 | Q52LR7 | enhancer of polycomb homolog 2 | 2.37 |
| 138 | Q96S55-1 | ATPase WRNIP1 | 2.36 |
| 139 | P23467 | Receptor-type tyrosine-protein phosphatase beta | 2.31 |
| 140 | Q9NX36 | DnaJ homolog subfamily C member 28 | 2.31 |
| 141 | P01715 | Immunoglobulin lambda variable 3-1 | 2.27 |
| 142 | Q28085 | Complement factor H GN=CFH PE=1 SV=3 | 2.22 |
| 143 | Q86VI1 | Exocyst complex component 3-like protein | 2.11 |
| 144 | A0A075B6I9 | Immunoglobulin lambda variable 7-46 | 2.1 |
| 145 | Q9ULD2-6 | Isoform 6 of Microtubule-associated tumor suppressor 1 | 2.06 |
| 146 | Q16777 | Histone H2A type 2-C | 2.05 |
| 147 | Q8WWM7-1 | ataxin-2-like protein | 2.04 |
| 148 | Q14247-1 | Src substrate cortactin | 2 |
| 149 | Q15697-2 | Isoform 2 of Zinc finger protein 174 | 2 |
| 150 | Q9UL12 | Sarcosine dehydrogenase, mitochondrial | 2 |
| 151 | Q16820 | Meprin A subunit beta | 1.98 |
| 152 | Q9BXU0 | Testis-expressed protein 12 | 1.98 |
| 153 | Q92878 | DNA repair protein Rad50 | 1.95 |
| 154 | P63267-1 | actin, gamma-enteric smooth muscle | 1.94 |
| 155 | Q6ZWH5-1 | Serine/threonine-protein kinase Nek10 | 1.94 |
| 156 | Q9Y283-1 | Inversin | 1.92 |
| 157 | Q9NQS7 | Inner centromere protein | 1.91 |

**Peptide List Batch n=2**

| **Accession** | **Description** | **Coverage [%]** | **# Peptides** | **Score Sequest HT** |
| --- | --- | --- | --- | --- |
| *P08603-1* | *complement factor H* | *69* | *75* | *1135.38* |
|  | Sequence |  |  | Score Sequest HT |
|  | AGEQVTYTCATYYK |  |  | 4.13 |
|  | AQTTVTCMENGWSPTPR |  |  | 3.68 |
|  | AQTTVTCMENGWSPTPR |  |  | 3.8 |
|  | AVYTCNEGYQLLGEINYR |  |  | 5.13 |
|  | CFEGFGIDGPAIAK |  |  | 2.97 |
|  | CFEGFGIDGPAIAKCLGEK |  |  | 4.09 |
|  | CGPPPPIDNGDITSFPLSVYAPASSVEYQCQNLYQLEGNK |  |  | 7.7 |
|  | CGPPPPIDNGDITSFPLSVYAPASSVEYQCQNLYQLEGNKR |  |  | 7.36 |
|  | CLHPCVISR |  |  | 1.99 |
|  | CNMGYEYSER |  |  | 3.36 |
|  | CNMGYEYSER |  |  | 2.63 |
|  | CTLKPCDYPDIK |  |  | 3.77 |
|  | CTSTGWIPAPR |  |  | 3.54 |
|  | CVEISCKSPDVINGSPISQK |  |  | 3.18 |
|  | CYFPYLENGYNQNYGR |  |  | 4.84 |
|  | DGEKVSVLCQENYLIQEGEEITCK |  |  | 3.15 |
|  | DGWSAQPTCIK |  |  | 2.43 |
|  | DTSCVNPPTVQNAYIVSR |  |  | 5.51 |
|  | ECDTDGWTNDIPICEVVK |  |  | 3.99 |
|  | EEYGHSEVVEYYCNPR |  |  | 5.02 |
|  | EFDHNSNIR |  |  | 2.7 |
|  | EGWIHTVCINGR |  |  | 2.95 |
|  | EKTKEEYGHSEVVEYYCNPR |  |  | 4.72 |
|  | EIMENYNIALR |  |  | 2.78 |
|  | EIMENYNIALR |  |  | 2.71 |
|  | EQVQSCGPPPELLNGNVK |  |  | 4.38 |
|  | FSCKPGFTIVGPNSVQCYHFGLSPDLPICK |  |  | 4.89 |
|  | FVCNSGYKIEGDEEMHCSDDGFWSK |  |  | 4.19 |
|  | GDAVCTESGWRPLPSCEEK |  |  | 4.69 |
|  | GEWVALNPLR |  |  | 2.42 |
|  | GEWVALNPLRK |  |  | 2.34 |
|  | GKEGWIHTVCINGR |  |  | 2.94 |
|  | HGGLYHENMR |  |  | 3.64 |
|  | HGGLYHENMR |  |  | 2.66 |
|  | KCYFPYLENGYNQNYGR |  |  | 4.32 |
|  | KEFDHNSNIR |  |  | 3.77 |
|  | KGEWVALNPLR |  |  | 3.7 |
|  | KGEWVALNPLRK |  |  | 3.84 |
|  | IDVHLVPDR |  |  | 2.38 |
|  | IDVHLVPDRK |  |  | 2.83 |
|  | IEGDEEMHCSDDGFWSK |  |  | 3.86 |
|  | IEGDEEMHCSDDGFWSK |  |  | 2.67 |
|  | LGYVTADGETSGSITCGK |  |  | 4.83 |
|  | LGYVTADGETSGSITCGKDGWSAQPTCIK |  |  | 4.2 |
|  | IIYKENER |  |  | 2.19 |
|  | IIYKENERFQYK |  |  | 2.83 |
|  | LSYTCEGGFR |  |  | 2.32 |
|  | IVSSAMEPDR |  |  | 2.24 |
|  | IVSSAMEPDREYHFGQAVR |  |  | 5.31 |
|  | IVSSAMEPDREYHFGQAVR |  |  | 4.06 |
|  | NDFTWFK |  |  | 2.38 |
|  | NGFYPATR |  |  | 2.52 |
|  | NGQWSEPPK |  |  | 2.24 |
|  | NTEILTGSWSDQTYPEGTQAIYK |  |  | 4.96 |
|  | RNTEILTGSWSDQTYPEGTQAIYK |  |  | 6.04 |
|  | RPCGHPGDTPFGTFTLTGGNVFEYGVK |  |  | 7.27 |
|  | RPYFPVAVGK |  |  | 3.1 |
|  | SCDIPVFMNAR |  |  | 3.22 |
|  | SCDIPVFMNAR |  |  | 2 |
|  | SCDNPYIPNGDYSPLR |  |  | 4.88 |
|  | SIDVACHPGYALPK |  |  | 4.74 |
|  | SLGNVIMVCR |  |  | 2.91 |
|  | SLGNVIMVCR |  |  | 2.41 |
|  | SITCIHGVWTQLPQCVAIDK |  |  | 5.79 |
|  | SITCIHGVWTQLPQCVAIDKLK |  |  | 5.12 |
|  | SPDVINGSPISQK |  |  | 3.29 |
|  | SPPEISHGVVAHMSDSYQYGEEVTYK |  |  | 7.54 |
|  | SSIDIENGFISESQYTYALK |  |  | 5.24 |
|  | SSNLIILEEHLK |  |  | 3.86 |
|  | SSQESYAHGTK |  |  | 2.44 |
|  | TDCLSLPSFENAIPMGEK |  |  | 3.78 |
|  | TDCLSLPSFENAIPMGEK |  |  | 2.31 |
|  | TGDEITYQCR |  |  | 3.17 |
|  | TGESVEFVCK |  |  | 3.26 |
|  | TGESVEFVCKR |  |  | 4.06 |
|  | TKEEYGHSEVVEYYCNPR |  |  | 6.29 |
|  | TKNDFTWFK |  |  | 2.3 |
|  | TTCWDGKLEYPTCAK |  |  | 3.45 |
|  | VSVLCQENYLIQEGEEITCK |  |  | 4.53 |
|  | VSVLCQENYLIQEGEEITCKDGR |  |  | 6.23 |
|  | WQSIPLCVEK |  |  | 2.24 |
|  | WSHPPSCIK |  |  | 2.22 |
|  | WSSPPQCEGLPCK |  |  | 2.57 |
|  | YYSYYCDEHFETPSGSYWDHIHCTQDGWSPAVPCLR |  |  | 5.39 |

**Batch N=3 (code:18)**

Tot: 185 proteins (95% confidence)

| N | Accession | Description | Score |
| --- | --- | --- | --- |
| 1 | P08603-1 | complement factor H | 1456.57 |
| 2 | P01024 | Complement C3 | 715.19 |
| 3 | P01031 | Complement C5 | 697.64 |
| 4 | P01023 | alpha-2-macroglobulin | 302.61 |
| 5 | P02751 | fibronectin | 239.67 |
| 6 | P02675 | Fibrinogen beta chain | 176.87 |
| 7 | P0C0L5 | Complement C4-B | 153.68 |
| 8 | P0C0L4-1 | Complement C4-A | 150.27 |
| 9 | P02679 | Fibrinogen gamma chain | 137.06 |
| 10 | P01871 | immunoglobulin heavy constant mu | 115.65 |
| 11 | P01834 | immunoglobulin kappa constant | 106.87 |
| 12 | P02768-1 | Serum albumin | 101.09 |
| 13 | P0DOX6 | immunoglobulin mu heavy chain | 93.2 |
| 14 | P13645 | Keratin, type I cytoskeletal 10 | 92.99 |
| 15 | P04114 | apolipoprotein B-100 | 89.83 |
| 16 | P0DOX7 | immunoglobulin kappa light chain | 87.78 |
| 17 | P00760 | Cationic trypsin PE=1 SV=3 | 84.81 |
| 18 | P02671-1 | Fibrinogen alpha chain | 80.87 |
| 19 | P01876 | immunoglobulin heavy constant alpha 1 | 71.21 |
| 20 | Q03591 | Complement factor H-related protein 1 | 64.68 |
| 21 | P04220 | Ig MU heavy chain disease protein | 61.23 |
| 22 | P04264 | Keratin, type II cytoskeletal 1 | 60.78 |
| 23 | P0DOY2 | immunoglobulin lambda constant 2 | 54.77 |
| 24 | P35527 | Keratin, type I cytoskeletal 9 | 47.02 |
| 25 | B9A064-1 | Immunoglobulin lambda-like polypeptide 5 | 42.36 |
| 26 | P01857 | Immunoglobulin heavy constant gamma 1 | 39.17 |
| 27 | P10909-1 | Clusterin | 32.98 |
| 28 | Q2UVX4 | Complement C3 GN=C3 PE=1 SV=2 | 31.53 |
| 29 | P00736 | Complement C1r subcomponent | 31.3 |
| 30 | Q02985-1 | Complement factor H-related protein 3 | 30.48 |
| 31 | P02769 | Serum albumin GN=ALB PE=1 SV=4 | 28.16 |
| 32 | P00751-1 | Complement factor B | 26.35 |
| 33 | P00747 | Plasminogen | 26.01 |
| 34 | P35858 | Insulin-like growth factor-binding protein complex acid labile subunit | 25.47 |
| 35 | P01860 | Immunoglobulin heavy constant gamma 3 | 24.97 |
| 36 | P01591 | Immunoglobulin J chain | 24.79 |
| 37 | Q14624-1 | Inter-alpha-trypsin inhibitor heavy chain H4 | 24.46 |
| 38 | P29622 | Kallistatin | 21.97 |
| 39 | O43866 | CD5 antigen-like | 21.74 |
| 40 | P19823 | Inter-alpha-trypsin inhibitor heavy chain H2 | 19.92 |
| 41 | P19827-1 | Inter-alpha-trypsin inhibitor heavy chain H1 | 19.63 |
| 42 | P35908 | Keratin, type II cytoskeletal 2 epidermal | 17.93 |
| 43 | P04196 | Histidine-rich glycoprotein | 16.84 |
| 44 | P01859 | Immunoglobulin heavy constant gamma 2 | 16.21 |
| 45 | P04259 | keratin, type II cytoskeletal 6B | 14.95 |
| 46 | P00748 | Coagulation factor XII | 14.87 |
| 47 | Q9UJJ9 | N-acetylglucosamine-1-phosphotransferase subunit gamma | 13.87 |
| 48 | P49908 | Selenoprotein P | 13.32 |
| 49 | P22105 | tenascin-X | 13.03 |
| 50 | P09871 | Complement C1s subcomponent | 12.87 |
| 51 | P07358 | Complement component C8 beta chain | 12.49 |
| 52 | P36955 | Pigment epithelium-derived factor | 12.11 |
| 53 | P13671 | Complement component c6 | 11.73 |
| 54 | P07357 | Complement component C8 alpha chain | 11.14 |
| 55 | P01009-1 | alpha-1-antitrypsin | 10.54 |
| 56 | P19652 | Alpha-1-acid glycoprotein 2 | 10.31 |
| 57 | P08779 | Keratin, type I cytoskeletal 16 | 9.89 |
| 58 | P05546 | Heparin cofactor 2 | 9.77 |
| 59 | Q9UK55 | Protein Z-dependent protease inhibitor | 8.84 |
| 60 | P68871 | Hemoglobin subunit beta | 8.61 |
| 61 | Q922U2 | Keratin, type II cytoskeletal 5 GN=Krt5 PE=1 SV=1 | 8.58 |
| 62 | P07744 | Keratin, type II cytoskeletal 4 GN=Krt4 PE=1 SV=2 | 8.4 |
| 63 | P00738 | Haptoglobin | 8.35 |
| 64 | P01780 | Immunoglobulin heavy variable 3-7 | 8.17 |
| 65 | Q13885 | Tubulin beta-2A chain | 7.81 |
| 66 | Q06033-1 | Inter-alpha-trypsin inhibitor heavy chain H3 | 7.68 |
| 67 | P03952 | Plasma kallikrein | 7 |
| 68 | P01019 | Angiotensinogen | 6.65 |
| 69 | Q92954-1 | Proteoglycan 4 | 6.62 |
| 70 | Q16777 | Histone H2A type 2-C | 6.43 |
| 71 | P05155 | Plasma protease C1 inhibitor | 6.33 |
| 72 | O60244 | Mediator of RNA polymerase II transcription subunit 14 | 6.07 |
| 73 | O15265 | Ataxin-7 | 6.03 |
| 74 | P23528 | Cofilin-1 | 5.83 |
| 75 | P02747 | Complement C1q subcomponent subunit C | 5.46 |
| 76 | Q96JB1-1 | Dynein heavy chain 8, axonemal | 5.34 |
| 77 | P0DP04 | Immunoglobulin heavy variable 3-43D | 4.83 |
| 78 | Q8IUR5-2 | Isoform 2 of Transmembrane and TPR repeat-containing protein 1 | 4.64 |
| 79 | P01619 | Immunoglobulin kappa variable 3-20 | 4.58 |
| 80 | P01011-1 | Alpha-1-antichymotrypsin | 4.51 |
| 81 | Q86X10-1 | Ral GTPase-activating protein subunit beta | 4.49 |
| 82 | Q5T011 | KICSTOR complex protein SZT2 | 4.48 |
| 83 | P07477 | Trypsin-1 | 4.38 |
| 84 | P69905 | Hemoglobin subunit alpha | 4.13 |
| 85 | P80748 | Immunoglobulin lambda variable 3-21 | 3.85 |
| 86 | P06331 | immunoglobulin heavy variable 4-34 | 3.74 |
| 87 | Q16778 | Histone H2B type 2-E | 3.58 |
| 88 | P60174-1 | Isoform 2 of Triosephosphate isomerase | 3.55 |
| 89 | Q96AD5-1 | Patatin-like phospholipase domain-containing protein 2 | 3.55 |
| 90 | P04004 | Vitronectin | 3.52 |
| 91 | Q86TB3 | Alpha-protein kinase 2 | 3.51 |
| 92 | P04406-1 | glyceraldehyde-3-phosphate dehydrogenase | 3.41 |
| 93 | O95147 | Dual specificity protein phosphatase 14 | 3.38 |
| 94 | P01700 | Immunoglobulin lambda variable 1-47 | 3.35 |
| 95 | Q99501 | GAS2-like protein 1 | 3.31 |
| 96 | P50607 | Tubby protein homolog | 3.27 |
| 97 | P02760 | Protein AMBP | 3.26 |
| 98 | Q9UMQ3 | Homeobox protein BarH-like 2 | 3.22 |
| 99 | A6NEQ2 | Protein FAM181B | 3.21 |
| 100 | O00267 | Transcription elongation factor Spt5 | 3.2 |
| 101 | Q9Y5H5-1 | Protocadherin alpha-9 | 3.16 |
| 102 | Q8NF91 | Nesprin-1 | 3.15 |
| 103 | P46379-1 | Large proline-rich protein BAG6 | 3.08 |
| 104 | P08572 | Collagen alpha-2(IV) chain | 3.05 |
| 105 | Q93034 | Cullin-5 | 2.98 |
| 106 | A8MPY1 | Gamma-aminobutyric acid receptor subunit rho-3 | 2.97 |
| 107 | Q5M775-1 | Cytospin-B | 2.96 |
| 108 | Q9P253 | Vacuolar protein sorting-associated protein 18 homolog | 2.96 |
| 109 | P08235 | mineralocorticoid receptor | 2.96 |
| 110 | P18858 | DNA ligase 1 | 2.96 |
| 111 | Q8N8A6 | ATP-dependent RNA helicase DDX51 | 2.95 |
| 112 | P42701 | Interleukin-12 receptor subunit beta-1 | 2.95 |
| 113 | Q9BV20-1 | methylthioribose-1-phosphate isomerase | 2.95 |
| 114 | Q01167 | Forkhead box protein K2 | 2.94 |
| 115 | Q96QE3-1 | ATPase family AAA domain-containing protein 5 | 2.89 |
| 116 | Q659A1-1 | Little elongation complex subunit 2 | 2.88 |
| 117 | Q9Y613 | FH1/FH2 domain-containing protein 1 | 2.86 |
| 118 | P01833 | Polymeric immunoglobulin receptor | 2.86 |
| 119 | P34932 | Heat shock 70 kDa protein 4 | 2.85 |
| 120 | Q8IUC4 | Rhophilin-2 | 2.84 |
| 121 | P78524-1 | Suppression of tumorigenicity 5 protein | 2.83 |
| 122 | Q9H7U1 | serine-rich coiled-coil domain-containing protein 2 | 2.83 |
| 123 | Q9ULL5-3 | Isoform 3 of Proline-rich protein 12 | 2.83 |
| 124 | P42345 | Serine/threonine-protein kinase mTOR | 2.82 |
| 125 | I1YAP6 | Tripartite motif-containing protein 77 | 2.81 |
| 126 | P61978 | Heterogeneous nuclear ribonucleoprotein K | 2.8 |
| 127 | Q9ULW0-2 | Isoform 2 of Targeting protein for Xklp2 | 2.79 |
| 128 | Q8IWV7-1 | E3 ubiquitin-protein ligase UBR1 | 2.79 |
| 129 | Q07507 | Dermatopontin | 2.79 |
| 130 | P02461 | Collagen alpha-1(III) chain | 2.79 |
| 131 | Q9BTC8 | Metastasis-associated protein MTA3 | 2.77 |
| 132 | Q7Z5P9 | Mucin-19 | 2.74 |
| 133 | Q86W34-4 | Archaemetzincin-2 | 2.71 |
| 134 | Q9Y2G1 | Myelin regulatory factor | 2.7 |
| 135 | Q9P2K6 | Kelch-like protein 42 | 2.7 |
| 136 | Q14693-1 | Phosphatidate phosphatase LPIN1 | 2.7 |
| 137 | Q8N7P7 | Uncharacterized protein FLJ40521 | 2.7 |
| 138 | Q8N5R6-6 | Isoform 6 of Coiled-coil domain-containing protein 33 | 2.69 |
| 139 | P06312 | immunoglobulin kappa variable 4-1 | 2.68 |
| 140 | O14683 | Tumor protein p53-inducible protein 11 | 2.68 |
| 141 | P50416 | Carnitine O-palmitoyltransferase 1, liver isoform | 2.68 |
| 142 | Q9BZ23 | Pantothenate kinase 2, mitochondrial | 2.66 |
| 143 | Q2TAM9 | Tumor suppressor candidate gene 1 protein | 2.66 |
| 144 | Q9BVQ7-1 | Spermatogenesis-associated protein 5-like protein 1 | 2.65 |
| 145 | Q13515 | Phakinin | 2.65 |
| 146 | Q6BDS2 | UHRF1-binding protein 1 | 2.65 |
| 147 | Q9NSC2 | Sal-like protein 1 | 2.65 |
| 148 | Q9UPQ7-1 | E3 ubiquitin-protein ligase PDZRN3 | 2.63 |
| 149 | P51817 | camp-dependent protein kinase catalytic subunit prkx | 2.63 |
| 150 | P29016 | T-cell surface glycoprotein CD1b | 2.62 |
| 151 | O94933 | SLIT and NTRK-like protein 3 | 2.62 |
| 152 | Q8TEW0 | Partitioning defective 3 homolog | 2.62 |
| 153 | Q08AM6 | Protein VAC14 homolog | 2.6 |
| 154 | A0A0A0MRZ8 | immunoglobulin kappa variable 3D-11 | 2.6 |
| 155 | P04003 | C4b-binding protein alpha chain | 2.57 |
| 156 | O75970 | multiple PDZ domain protein | 2.57 |
| 157 | Q5SNV9 | Uncharacterized protein C1orf167 | 2.56 |
| 158 | P23467 | Receptor-type tyrosine-protein phosphatase beta | 2.55 |
| 159 | Q8N944 | APC membrane recruitment protein 3 | 2.54 |
| 160 | Q15582 | Transforming growth factor-beta-induced protein ig-h3 | 2.49 |
| 161 | Q9C0H6-1 | kelch-like protein 4 | 2.48 |
| 162 | Q7L590-1 | Protein MCM10 homolog | 2.46 |
| 163 | A0A0C4DH38 | immunoglobulin heavy variable 5-51 | 2.44 |
| 164 | O95425 | Supervillin | 2.42 |
| 165 | Q13748-1 | tubulin alpha-3C/D chain | 2.41 |
| 166 | Q5HYC2-1 | Uncharacterized protein KIAA2026 | 2.4 |
| 167 | Q8WWM7-1 | ataxin-2-like protein | 2.39 |
| 168 | P25054-1 | Adenomatous polyposis coli protein | 2.35 |
| 169 | Q9NXC5-1 | GATOR complex protein MIOS | 2.34 |
| 170 | Q8N2H3 | Pyridine nucleotide-disulfide oxidoreductase domain-containing protein 2 | 2.33 |
| 171 | Q8TAX0 | Protein odd-skipped-related 1 | 2.33 |
| 172 | P35659-1 | Protein DEK | 2.31 |
| 173 | Q86VI1 | Exocyst complex component 3-like protein | 2.3 |
| 174 | Q28085 | Complement factor H GN=CFH PE=1 SV=3 | 2.28 |
| 175 | P49643-1 | DNA primase large subunit | 2.12 |
| 176 | Q53F19 | Nuclear cap-binding protein subunit 3 | 2.1 |
| 177 | Q9ULD2-6 | Isoform 6 of Microtubule-associated tumor suppressor 1 | 2.05 |
| 178 | Q14247-1 | Src substrate cortactin | 2.04 |
| 179 | Q9NRY6 | Phospholipid scramblase 3 | 2.03 |
| 180 | P07225 | Vitamin K-dependent protein S | 2.03 |
| 181 | P26358-1 | DNA (cytosine-5)-methyltransferase 1 | 2.01 |
| 182 | Q92878 | DNA repair protein Rad50 | 1.98 |
| 183 | P53396-1 | ATP-citrate synthase | 1.98 |
| 184 | P02649 | Apolipoprotein E | 1.96 |
| 185 | Q969Z0-1 | Protein TBRG4 | 1.93 |

**Peptide List Batch n=3**

| **Accession** | **Description** | **Coverage [%]** | **# Peptides** | **Score Sequest HT** |
| --- | --- | --- | --- | --- |
| *P08603-1* | *complement factor H* | *70* | *84* | *1456.57* |
|  | Sequence |  |  | Score Sequest HT |
|  | AGEQVTYTCATYYK |  |  | 4.08 |
|  | AQTTVTCMENGWSPTPR |  |  | 3.11 |
|  | AVYTCNEGYQLLGEINYR |  |  | 5.19 |
|  | CFEGFGIDGPAIAK |  |  | 2.77 |
|  | CFEGFGIDGPAIAKCLGEK |  |  | 4.01 |
|  | CGPPPPIDNGDITSFPLSVYAPASSVEYQCQNLYQLEGNK |  |  | 8.59 |
|  | CGPPPPIDNGDITSFPLSVYAPASSVEYQCQNLYQLEGNKR |  |  | 8.83 |
|  | CLGEKWSHPPSCIK |  |  | 3.41 |
|  | CLHPCVISR |  |  | 2.01 |
|  | CLPVTAPENGK |  |  | 2.59 |
|  | CNMGYEYSER |  |  | 3.36 |
|  | CNMGYEYSER |  |  | 2.53 |
|  | CTLKPCDYPDIK |  |  | 3.6 |
|  | CTSTGWIPAPR |  |  | 3.49 |
|  | CVEISCK |  |  | 2.01 |
|  | CVEISCKSPDVINGSPISQK |  |  | 4.31 |
|  | CYFPYLENGYNQNYGR |  |  | 4.94 |
|  | DGEKVSVLCQENYLIQEGEEITCK |  |  | 5.39 |
|  | DGWSAQPTCIK |  |  | 2.45 |
|  | DTSCVNPPTVQNAYIVSR |  |  | 5.54 |
|  | ECDTDGWTNDIPICEVVK |  |  | 4.11 |
|  | ECELPKIDVHLVPDR |  |  | 3.27 |
|  | EEYGHSEVVEYYCNPR |  |  | 5.23 |
|  | EFDHNSNIR |  |  | 2.78 |
|  | EGWIHTVCINGR |  |  | 2.92 |
|  | EKTKEEYGHSEVVEYYCNPR |  |  | 5.6 |
|  | EIMENYNIALR |  |  | 2.77 |
|  | EIMENYNIALR |  |  | 2.37 |
|  | EQVQSCGPPPELLNGNVK |  |  | 4.59 |
|  | FSCKPGFTIVGPNSVQCYHFGLSPDLPICK |  |  | 4.83 |
|  | FVCNSGYKIEGDEEMHCSDDGFWSK |  |  | 5.1 |
|  | GDAVCTESGWRPLPSCEEK |  |  | 4.42 |
|  | GEWVALNPLR |  |  | 2.39 |
|  | GEWVALNPLRK |  |  | 2.67 |
|  | GKEGWIHTVCINGR |  |  | 4.01 |
|  | HGGLYHENMR |  |  | 3.01 |
|  | HGGLYHENMR |  |  | 1.98 |
|  | HRTGDEITYQCR |  |  | 3.28 |
|  | KCYFPYLENGYNQNYGR |  |  | 4.68 |
|  | KEFDHNSNIR |  |  | 3.75 |
|  | KGEWVALNPLR |  |  | 4.07 |
|  | KGEWVALNPLRK |  |  | 4.04 |
|  | IDVHLVPDR |  |  | 2.46 |
|  | IDVHLVPDRK |  |  | 3.07 |
|  | IEGDEEMHCSDDGFWSK |  |  | 4.57 |
|  | IEGDEEMHCSDDGFWSK |  |  | 2.92 |
|  | LGYVTADGETSGSITCGK |  |  | 4.72 |
|  | LGYVTADGETSGSITCGKDGWSAQPTCIK |  |  | 5.74 |
|  | IIYKENER |  |  | 2.12 |
|  | IIYKENERFQYK |  |  | 2.82 |
|  | LSYTCEGGFR |  |  | 2.29 |
|  | IVSSAMEPDR |  |  | 2.42 |
|  | IVSSAMEPDREYHFGQAVR |  |  | 4.87 |
|  | IVSSAMEPDREYHFGQAVR |  |  | 4.75 |
|  | NDFTWFK |  |  | 2.34 |
|  | NGFYPATR |  |  | 2.41 |
|  | NGQWSEPPK |  |  | 2.26 |
|  | NTEILTGSWSDQTYPEGTQAIYK |  |  | 4.63 |
|  | RNTEILTGSWSDQTYPEGTQAIYK |  |  | 6.48 |
|  | RPCGHPGDTPFGTFTLTGGNVFEYGVK |  |  | 7.69 |
|  | RPCGHPGDTPFGTFTLTGGNVFEYGVK |  |  | 5.73 |
|  | RPYFPVAVGK |  |  | 3.63 |
|  | SCDIPVFMNAR |  |  | 3.21 |
|  | SCDIPVFMNAR |  |  | 1.98 |
|  | SCDNPYIPNGDYSPLR |  |  | 4.66 |
|  | SLGNVIMVCR |  |  | 3.21 |
|  | SLGNVIMVCR |  |  | 2.43 |
|  | SITCIHGVWTQLPQCVAIDK |  |  | 5.1 |
|  | SITCIHGVWTQLPQCVAIDKLK |  |  | 4.88 |
|  | SPDVINGSPISQK |  |  | 2.6 |
|  | SPDVINGSPISQKIIYK |  |  | 4.98 |
|  | SPPEISHGVVAHMSDSYQYGEEVTYK |  |  | 8.63 |
|  | SPPEISHGVVAHMSDSYQYGEEVTYK |  |  | 7.38 |
|  | SSIDIENGFISESQYTYALK |  |  | 5.11 |
|  | SSNLIILEEHLK |  |  | 4.12 |
|  | SSNLIILEEHLKNKK |  |  | 4.6 |
|  | SSQESYAHGTK |  |  | 2.93 |
|  | TDCLSLPSFENAIPMGEK |  |  | 3.93 |
|  | TDCLSLPSFENAIPMGEK |  |  | 2.98 |
|  | TGDEITYQCR |  |  | 3.48 |
|  | TGESVEFVCK |  |  | 3.34 |
|  | TGESVEFVCKR |  |  | 3.93 |
|  | TKEEYGHSEVVEYYCNPR |  |  | 6.24 |
|  | TKNDFTWFK |  |  | 2.89 |
|  | TTCWDGK |  |  | 1.92 |
|  | TTCWDGKLEYPTCAK |  |  | 3.73 |
|  | VSVLCQENYLIQEGEEITCK |  |  | 4.36 |
|  | VSVLCQENYLIQEGEEITCKDGR |  |  | 5.82 |
|  | WQSIPLCVEK |  |  | 2.24 |
|  | WSHPPSCIK |  |  | 2.59 |
|  | WSSPPQCEGLPCK |  |  | 3.88 |
|  | WTGRPTCR |  |  | 1.94 |
|  | WTGRPTCRDTSCVNPPTVQNAYIVSR |  |  | 4.88 |
|  | YYSYYCDEHFETPSGSYWDHIHCTQDGWSPAVPCLR |  |  | 5.92 |

**Table S3:** list of common proteins in the analyzed batches

|  |  | *Number of common proteins* | | | |
| --- | --- | --- | --- | --- | --- |
|  |  | *87* | *123* | *94* | *94* |
| **Accession** | **Description** | **Common All** | **Common 1,2** | **Common 1,3** | **Common 2,3** |
| P08603-1 | complement factor H | X | X | X | X |
| P01024 | Complement C3 | X | X | X | X |
| P0C0L5 | Complement C4-B | X | X | X | X |
| P0C0L4-1 | Complement C4-A | X | X | X | X |
| P01031 | Complement C5 | X | X | X | X |
| P02751 | fibronectin | X | X | X | X |
| P01023 | alpha-2-macroglobulin | X | X | X | X |
| P01871 | immunoglobulin heavy constant mu | X | X | X | X |
| P04220 | Ig MU heavy chain disease protein | X | X | X | X |
| P04264 | Keratin, type II cytoskeletal 1 | X | X | X | X |
| P01834 | immunoglobulin kappa constant | X | X | X | X |
| P02768-1 | Serum albumin | X | X | X | X |
| P02675 | Fibrinogen beta chain | X | X | X | X |
| P13645 | Keratin, type I cytoskeletal 10 | X | X | X | X |
| P04114 | apolipoprotein B-100 | X | X | X | X |
| P0DOX7 | immunoglobulin kappa light chain | X | X | X | X |
| P35527 | Keratin, type I cytoskeletal 9 | X | X | X | X |
| P02679 | Fibrinogen gamma chain | X | X | X | X |
| P35908 | Keratin, type II cytoskeletal 2 epidermal | X | X | X | X |
| O43866 | CD5 antigen-like | X | X | X | X |
| P01876 | immunoglobulin heavy constant alpha 1 | X | X | X | X |
| P0DOY2 | immunoglobulin lambda constant 2 | X | X | X | X |
| P00760 | Cationic trypsin PE=1 SV=3 | X | X | X | X |
| P02671-1 | Fibrinogen alpha chain | X | X | X | X |
| P0DOX8 | Immunoglobulin lambda-1 light chain |  | X |  |  |
| P04003 | C4b-binding protein alpha chain | X | X | X | X |
| P00736 | Complement C1r subcomponent | X | X | X | X |
| P00747 | Plasminogen | X | X | X | X |
| P00751-1 | Complement factor B | X | X | X | X |
| Q03591 | Complement factor H-related protein 1 | X | X | X | X |
| P08779 | Keratin, type I cytoskeletal 16 |  |  | X |  |
| Q5XQN5 | Keratin, type II cytoskeletal 5 GN=KRT5 PE=1 SV=1 |  | X |  |  |
| Q922U2 | Keratin, type II cytoskeletal 5 GN=Krt5 PE=1 SV=1 |  |  | X |  |
| P01591 | Immunoglobulin J chain | X | X | X | X |
| P02769 | Serum albumin GN=ALB PE=1 SV=4 | X | X | X | X |
| P01860 | Immunoglobulin heavy constant gamma 3 | X | X | X | X |
| P00739-1 | Haptoglobin-related protein |  | X |  |  |
| P09871 | Complement C1s subcomponent | X | X | X | X |
| P07357 | Complement component C8 alpha chain | X | X | X | X |
| Q02985-1 | Complement factor H-related protein 3 | X | X | X | X |
| P19827-1 | Inter-alpha-trypsin inhibitor heavy chain H1 | X | X | X | X |
| P10909-1 | Clusterin | X | X | X | X |
| P19823 | Inter-alpha-trypsin inhibitor heavy chain H2 | X | X | X | X |
| P04004 | Vitronectin | X | X | X | X |
| Q14624-1 | Inter-alpha-trypsin inhibitor heavy chain H4 | X | X | X | X |
| P00738 | Haptoglobin | X | X | X | X |
| P01833 | Polymeric immunoglobulin receptor | X | X | X | X |
| P35858 | Insulin-like growth factor-binding protein complex acid labile subunit | X | X | X | X |
| P0DOX2 | Immunoglobulin alpha-2 heavy chain |  | X |  |  |
| P02748 | complement component C9 |  | X |  |  |
| P05546 | Heparin cofactor 2 | X | X | X | X |
| A0A0C4DH72 | immunoglobulin kappa variable 1-6 |  | X |  |  |
| P01859 | Immunoglobulin heavy constant gamma 2 | X | X | X | X |
| P01619 | Immunoglobulin kappa variable 3-20 | X | X | X | X |
| P29622 | Kallistatin | X | X | X | X |
| P22105 | tenascin-X | X | X | X | X |
| A0A0B4J1X5 | immunoglobulin heavy variable 3-74 |  | X |  |  |
| P27918 | Properdin |  | X |  |  |
| P04196 | Histidine-rich glycoprotein | X | X | X | X |
| P03952 | Plasma kallikrein | X | X | X | X |
| P01009-1 | alpha-1-antitrypsin | X | X | X | X |
| P49908 | Selenoprotein P | X | X | X | X |
| P81605 | Dermcidin |  | X |  |  |
| P07360 | Complement component C8 gamma chain |  | X |  |  |
| P07358 | Complement component C8 beta chain | X | X | X | X |
| P19652 | Alpha-1-acid glycoprotein 2 | X | X | X | X |
| P27169 | Serum paraoxonase/arylesterase 1 |  | X |  |  |
| P01780 | Immunoglobulin heavy variable 3-7 | X | X | X | X |
| P06331 | immunoglobulin heavy variable 4-34 | X | X | X | X |
| P68871 | Hemoglobin subunit beta | X | X | X | X |
| P02790 | Hemopexin |  | X |  |  |
| P02760 | Protein AMBP | X | X | X | X |
| P01701 | immunoglobulin lambda variable 1-51 |  | X |  |  |
| A0A075B6S5 | immunoglobulin kappa variable 1-27 |  | X |  |  |
| Q13885 | Tubulin beta-2A chain | X | X | X | X |
| P50607 | Tubby protein homolog | X | X | X | X |
| P01599 | Immunoglobulin kappa variable 1-17 |  | X |  |  |
| P02747 | Complement C1q subcomponent subunit C | X | X | X | X |
| A0A0A0MRZ8 | immunoglobulin kappa variable 3D-11 | X | X | X | X |
| P80748 | Immunoglobulin lambda variable 3-21 | X | X | X | X |
| P00748 | Coagulation factor XII | X | X | X | X |
| O75636-1 | Ficolin-3 |  | X |  |  |
| P01042 | kininogen-1 |  | X |  |  |
| P13671 | Complement component c6 | X | X | X | X |
| A0A0C4DH38 | immunoglobulin heavy variable 5-51 | X | X | X | X |
| P06312 | immunoglobulin kappa variable 4-1 | X | X | X | X |
| P01700 | Immunoglobulin lambda variable 1-47 | X | X | X | X |
| Q9P2C4 | Transmembrane protein 181 |  | X |  |  |
| P0DP04 | Immunoglobulin heavy variable 3-43D | X | X | X | X |
| Q9UK55 | Protein Z-dependent protease inhibitor | X | X | X | X |
| A0A0A0MS15 | immunoglobulin heavy variable 3-49 |  | X |  |  |
| Q16777 | Histone H2A type 2-C | X | X | X | X |
| Q92954-1 | Proteoglycan 4 | X | X | X | X |
| P01011-1 | Alpha-1-antichymotrypsin | X | X | X | X |
| P06396 | Gelsolin |  | X |  |  |
| P07225 | Vitamin K-dependent protein S | X | X | X | X |
| P02649 | Apolipoprotein E | X | X | X | X |
| P01019 | Angiotensinogen | X | X | X | X |
| P04430 | Immunoglobulin kappa variable 1-16 |  | X |  |  |
| Q86X10-1 | Ral GTPase-activating protein subunit beta |  |  | X |  |
| A0A0B4J1U7 | immunoglobulin heavy variable 6-1 |  | X |  |  |
| A0A0B4J2D9 | immunoglobulin kappa variable 1D-13 |  | X |  |  |
| A0A0C4DH68 | immunoglobulin kappa variable 2-24 |  | X |  |  |
| P69905 | Hemoglobin subunit alpha | X | X | X | X |
| Q8N8A6 | ATP-dependent RNA helicase DDX51 | X | X | X | X |
| P05155 | Plasma protease C1 inhibitor | X | X | X | X |
| P01594 | Immunoglobulin kappa variable 1-33 |  | X |  |  |
| A0A0B4J1V0 | Immunoglobulin heavy variable 3-15 |  | X |  |  |
| P00973-1 | 2'-5'-oligoadenylate synthase 1 |  | X |  |  |
| Q01167 | Forkhead box protein K2 |  |  | X |  |
| Q9BV20-1 | methylthioribose-1-phosphate isomerase |  |  | X |  |
| A0A0A0MT36 | Immunoglobulin kappa variable 6D-21 |  | X |  |  |
| Q53F19 | Nuclear cap-binding protein subunit 3 | X | X | X | X |
| A0A075B6I1 | immunoglobulin lambda variable 4-60 |  | X |  |  |
| Q9H7U1 | serine-rich coiled-coil domain-containing protein 2 |  |  | X |  |
| Q8N7P7 | Uncharacterized protein FLJ40521 |  |  | X |  |
| Q8WWM7-1 | ataxin-2-like protein | X | X | X | X |
| Q28085 | Complement factor H GN=CFH PE=1 SV=3 | X | X | X | X |
| P02745 | Complement C1q subcomponent subunit A |  | X |  |  |
| P01714 | Immunoglobulin lambda variable 3-19 |  | X |  |  |
| P02765 | Alpha-2-HS-glycoprotein |  | X |  |  |
| Q9Y283-1 | Inversin |  | X |  |  |
| Q14247-1 | Src substrate cortactin | X | X | X | X |
| P07477 | Trypsin-1 | X | X | X | X |
| Q15582 | Transforming growth factor-beta-induced protein ig-h3 | X | X | X | X |
| A0A075B6I9 | Immunoglobulin lambda variable 7-46 |  | X |  |  |
| Q9ULD2-6 | Isoform 6 of Microtubule-associated tumor suppressor 1 | X | X | X | X |
| Q6ZWH5-1 | Serine/threonine-protein kinase Nek10 |  | X |  |  |
| Q9BXU0 | Testis-expressed protein 12 |  | X |  |  |
| Q06033-1 | Inter-alpha-trypsin inhibitor heavy chain H3 | X | X | X | X |
| P01857 | Immunoglobulin heavy constant gamma 1 |  |  |  | X |
| P04259 | keratin, type II cytoskeletal 6B |  |  |  | X |
| Q5SNV9 | Uncharacterized protein C1orf167 |  |  |  | X |
| Q86TB3 | Alpha-protein kinase 2 |  |  |  | X |
| P23467 | Receptor-type tyrosine-protein phosphatase beta |  |  |  | X |
| Q86VI1 | Exocyst complex component 3-like protein |  |  |  | X |
| Q92878 | DNA repair protein Rad50 |  |  |  | X |
